# TREM2 macrophage promotes cardiac repair in myocardial infarction by reprogramming metabolism via SLC25A53

**DOI:** 10.1038/s41418-023-01252-8

**Published:** 2024-01-05

**Authors:** Shiyu Gong, Ming Zhai, Jiayun Shi, Guanye Yu, Zhijun Lei, Yefei Shi, Yanxi Zeng, Peinan Ju, Na Yang, Zhuo Zhang, Donghui Zhang, Jianhui Zhuang, Qing Yu, Xumin Zhang, Weixia Jian, Wei Wang, Wenhui Peng

**Affiliations:** 1 Department of Cardiology, Shanghai Tenth People’s Hospital, Tongji University, School of Medicine, Shanghai, China; 2 State Key Laboratory of Biocatalysis and Enzyme Engineering, School of Life Science, Hubei University, Wuhan, 430062 China; 3 Institute of Cardiovascular Diseases, Union Hospital, Tongji Medical College, Huazhong University of Science and Technology, Wuhan, 430022 China; 4 Pan-Vascular Research Institute of Tongji University, Shanghai Tenth People’s Hospital, Tongji University School of Medicine, 301 Middle Yanchang Road, Shanghai, 200072 China; 5 Department of Cardiology, Shanghai East Hospital, Tongji University, School of Medicine, Shanghai, China; 6 Department of Endocrinology, Xinhua Hospital, Shanghai Jiaotong University, School of Medicine, Shanghai, China

**Keywords:** Immunology, Cardiovascular diseases, Metabolomics, Small molecules

## Abstract

Efferocytosis and metabolic reprogramming of macrophages play crucial roles in myocardial infarction (MI) repair. TREM2 has been proven to participate in phagocytosis and metabolism, but how it modulates myocardial infarction remains unclear. In this study, we showed that macrophage-specific TREM2 deficiency worsened cardiac function and impaired post-MI repair. Using RNA-seq, protein and molecular docking, and Targeted Metabolomics (LC–MS), our data demonstrated that macrophages expressing TREM2 exhibited decreased SLC25A53 transcription through the SYK-SMAD4 signaling pathway after efferocytosis, which impaired NAD^+^ transport into mitochondria, downregulated SLC25A53 thereby causing the breakpoint in the TCA cycle and subsequently increased itaconate production. In vitro experiments confirmed that itaconate secreted by TREM2^+^ macrophages inhibited cardiomyocyte apoptosis and promoted fibroblast proliferation. Conversely, overexpression of TREM2 in macrophages could improve cardiac function. In summary, our study reveals a novel role for macrophage-specific TREM2 in MI, connecting efferocytosis to immune metabolism during cardiac repair.

## Introduction

With the advancements in revascularization therapy and medical treatment, myocardial infarction (MI) patients have experienced significant benefits, but MI continues to be the leading cause of death worldwide [1]. In the acute stage, cardiac shock poses a threat, while heart failure due to cardiac remodeling becomes a concern in the chronic stage. Various post-reperfusion medications which prevent long-term ventricular remodeling have led to a substantial delay in disease progression and an improvement in patients’ quality of life. Nonetheless, therapeutic interventions targeting dead cell clearance and cardiac tissue healing remain limited. It is well-known that the healing process of MI needs the timely involvement of monocytes/macrophages, which scavenge dead cardiomyocytes and maintain a balance between pro-inflammatory and anti-inflammatory factors in the local myocardium [2].

Cardiac macrophages play a crucial role in the healing process of myocardial infarction. These macrophages can originate from the resident population within the cardiac tissue or be recruited from the circulating monocyte to the ischemic heart tissue. They possess the ability to sense the local environment, engulf dead cellular components, undergo metabolic changes, and ultimately contribute to healing [3]. Numerous molecules have been reported to be involved in these processes by regulating the function of different macrophage subsets [4]. Incomplete clearance of dying cells and persistent inflammation following myocardial infarction result in adverse cardiac remodeling and dysfunction that lead to heart failure. Thus, we believe that targeting macrophages might bring additional benefits to heart function recovery and become the next milestone for ischemic heart disease treatment.

In recent years, several studies have distinguished a distinct subpopulation of macrophages that express high levels of Triggering Receptor Expressed on Myeloid Cells 2 (TREM2) [5–7]. Upon ligand binding, TREM2 signals transduces through the adapter DAP12, thereby modulating activation of macrophages [8]. TREM2 is involved in regulating multiple cellular responses, including efferocytosis, as well as cell survival and inflammation [8]. TREM2 has been intensively studied in the context of neurodegenerative diseases, revealing its concurrent role in engulfment of Aβ-amyloid plaques and sustainment of cellular energetic and biosynthetic metabolism during Alzheimer’s disease [9]. In addition, TREM2 is involved in the regulatory processes in cancer, obesity, atherosclerosis, and liver disease [10–12]. However, previous studies have predominantly focused on the involvement of TREM2 in chronic diseases, whereas its role in acute disease settings such as MI remains unclear.

In this study, we aimed to explore the involvement of TREM2 in MI and elucidate its function in the healing process. Specifically, we observed that TREM2 influences the macrophage efferocytosis-glycometabolism and anti-inflammation pathway, which was essential for effective healing. Notably, we found that overexpression of TREM2 in macrophages can ameliorate the deterioration of cardiac function following MI, suggesting a promising therapeutic approach worthy of further investigation.

## Results

### Macrophages were the main TREM2-expressing cells

By screening different tissues, we found that TREM2 was highly expressed in the spleen, and bone marrow, consistent with reports from others (Supplementary Fig. 1A) [13]. In contrast, normal heart tissue had a relatively low TREM2 expression level. Using different cell lines, we identified BMDMs had the highest expression of TREM2 compared with cardiomyocytes, fibroblasts, and endothelial cells (Supplementary Fig. 1B). A further experiment focusing on immune cells demonstrated that BMDMs expressed a significantly higher level of TREM2 compared with other immunological cells (Supplementary Fig. 1C). RNA-seq of peripheral leukocytes showed that, besides macrophages, TREM2 was also expressed in dendritic cells and monocytes (Supplementary Fig. 1D). As a result, we confirmed that macrophages were the main cells expressing TREM2.

### TREM2^+^ macrophages were involved in the acute phase of MI

**Fig. 1:**
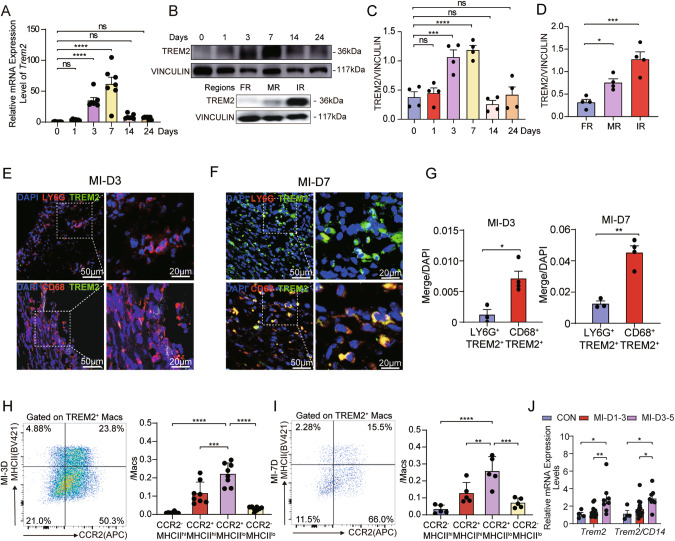
TREM2 is mainly expressed in immune tissues, and MI challenge induced TREM2 expression. **A** TREM2 expression was analyzed by quantitative polymerase chain reaction (qPCR) in murine heart on multi-day points post-MI (*n* = 7). **B** TREM2 expression was analyzed by western blot (WB) in murine heart on multi-day points post-MI (up) and in far region (FR), marginal region (MR) and ischemic region (IR) post-MI (down). **C**, **D** Related western blot analysis of **B** respectively (*n* = 4). **E**–**G** Representative immunofluorescence staining and related quantification of TREM2 and LY6G (up) and TREM2 andCD68 (down) in control murine heart on day 3 and day 7 post-MI (*n* = 3–4). **H**, **I** Gated strategy of TREM2^+^ macrophages’ subsets and related quantification on day 3 and day 7 post-MI (*n* = 5–8). **J** Relative mRNA expression level of TREM2 in peripheral blood mononuclear cells (PBMCs) extracted from MI patients (*n* = 14–15) and healthy volunteers (*n* = 10). Data were expressed as mean ± SEM. Data in **G** was analyzed by Mann–Whitney U tests. Other data were analyzed by one-way ANOVA followed by Bonferroni post hoc analysis. NS indicates not significant. ns indicates not significant. *P < 0.05. **P < 0.01. ***P < 0.001.

To elucidate the involvement of TREM2 in MI injury, we first examined the expression of TREM2 in the heart after MI. The expression level of TREM2 in MI hearts was significantly higher than the sham, suggesting the induction of TREM2 expression in response to ischemic challenge (Fig. 1A–C). It was worth noting that the expression of TREM2 exhibited a temporal pattern following MI. The expression levels began to increase on day 1 post-MI, showed a significant elevation on day 3, reached a peak on day 7, and eventually returned to baseline thereafter (Fig. 1A–C). The number of TREM2^+^ macrophages also increased after MI (Supplementary Fig. 2A). Further investigation of TREM2 expression in different regions of MI heart revealed that it was mainly expressed in the infarct and marginal region rather than the region distinct from the infarction or sham myocardium (Fig. 1B, D, and Supplementary Fig. 2B). Moreover, the majority of TREM2^+^ cells were CD68^+^ macrophages, rather than Ly6G^+^ neutrophils or CD11C^+^ dendritic cells (Fig. 1E–G and Supplementary Fig. 2C). Considering the transit recruitment of TREM2^+^ neutrophils infiltrating ischemic cardiac tissue compared to the relatively longer presence of TREM2^+^ macrophages during post-MI repair [14], we decided to focus our study on TREM2^+^ macrophages in MI.

The first question came through is whether the increase of TREM2 post-MI is related to the overexpression in macrophages or the number of macrophages. To clarify this question, we have quantified the expression of TREM2 in healthy heart of mice and those on day 7 post-MI by immunofluorescence per TREM2^+^ macrophage. Under the same shooting conditions, TREM2 fluorescence level and TREM2^+^ macrophages have significantly increased (Supplementary Fig. 3A). Moreover, we isolated macrophages from heart of mice from sham group and on day 7 post-myocardial infarction surgery. Subsequently, we assessed the RNA levels of TREM2 in comparison to CD68, a widely recognized marker for macrophages (Supplementary Fig. 3B). The result shows that there is significant change of TREM2 expression level within macrophages post-MI. In vitro, the expression of TREM2 also increased after RAW264.7 cells were stimulated by the supernatant of necrotic myocardial cells in vitro (Supplementary Fig. 3C, D). Thus, TREM2 expression level in macrophages and the number of TREM2 positive macrophages were all elevated. To further understand which subgroup of macrophages is the source of increased TREM2, we divided macrophages in heart as cardiac macrophages were mainly divided into three subgroups according to different biomarkers [15]. In WT mice, TREM2^+^ macrophages were predominantly CCR2^+^MHCII^low^ cells after MI (Fig. 1H, I for day 3 and 7, Supplementary Fig. 3E for day 1, and 14, with gating strategy described in Supplementary Fig. 3F), indicating that TREM2^+^ macrophages tended to exhibit an anti-inflammatory phenotype. In addition, we observed the expression levels of TREM2 in peripheral blood mononuclear cells (PBMCs) obtained from MI patients. Interestingly, we found that the levels of TREM2 were elevated in MI patients compared to healthy individuals, with higher levels observed on day 3–5 compared to day 1–3. Importantly, this increase in TREM2 expression was not attributed to the increase in the number of circulatory monocytes, as we confirmed by normalizing the expression levels to human monocyte CD14 expression (Fig. 1J). These findings suggested that the elevation of TREM2 expression in PBMCs of MI patients was likely due to the induction of TREM2 expression rather than changes in monocyte population.

### Macrophage-specific TREM2 defect aggravated MI injury

**Fig. 2:**
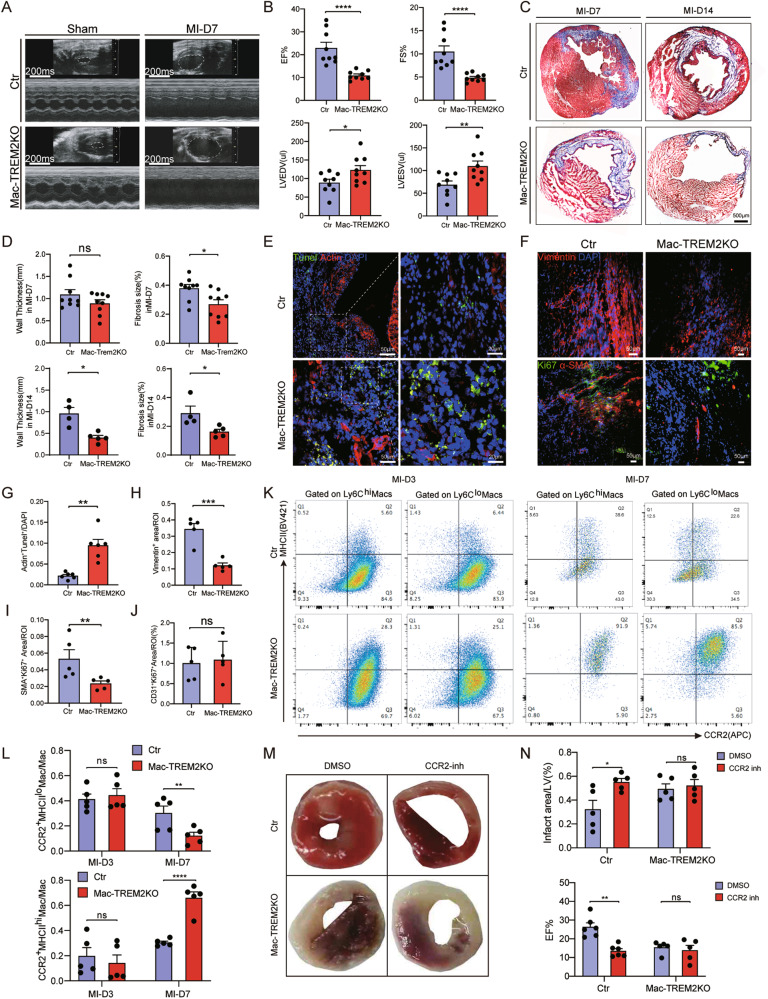
Effect of the TREM2’s conditional knockout in macrophages on the heart of MI mouse. **A** Representative M-mode echocardiogram images obtained from control and Mac-TREM2KO mice in MI or sham groups. **B** Echocardiographic analysis of ejection fraction (EF), fractional shortening (FS), left ventricular end-diastolic volume (LVEDV) and left ventricular end-systolic volume (LVESV) in groups described in **A** (*n* = 9 for each group). **C**, **D** Representative images of Masson’s trichrome staining and related quantification of left ventricular wall thickness and fibrosis area of control and Mac-TREM2KO mice on day 7 and 14 post-MI (*n* = 4–9). **E** Representative immunofluorescence images of TUNEL^+^ Actin^+^ cells in hearts of control and Mac-TREM2KO mice post-MI. **F** Representative immunofluorescence images of fibroblasts (Vimentin^+^ cells) and proliferated myofibroblasts (Ki67^+^ αSMA^+^ cells) in hearts of control and Mac-TREM2KO mice post-MI. **G-I** Relative quantification of **E** and **F** respectively (*n* = 5–6). **J**. Relative quantification of angiogenesis (Supplementary Fig. 4E, 5E) in hearts of control and Mac-TREM2KO mice post-MI (n = 5). **K**, **L**, Gated strategy and related quantification of macrophages in hearts of control and Mac-TREM2KO mice on day 3 and day 7 post-MI (*n* = 5). **M** Left ventricular tissue sections of control and Mac-TREM2KO mice administered with DMSO or CCR2 inhibitor were stained with 2,3,5-triphenyltetrazolium chloride on day 7 post-MI. **N** The ratios of infarct area/LV and echocardiographic analysis of groups described in **M** (*n* = 5). Data were expressed as mean ± SEM. Data in L and N were analyzed by 2-way ANOVA followed by Bonferroni post hoc analysis. Other data were analyzed by Mann–Whitney U tests. ns indicates not significant. *P < 0.05. **P < 0.01. ***P < 0.001.

To investigate the specific impact of TREM2^+^ macrophages on MI, we generated a conditional knockout of TREM2 in macrophages by crossbreeding LysM^Cre^ mice with TREM2^flox/flox^ mice (LysM^Cre^TREM2^flox/flox^ [Mac-TREM2KO]) and compared their response to MI with control mice (TREM2^flox/flox^). The efficiency of TREM2 knockout in macrophages was confirmed by qPCR (Supplementary Fig. 4A, B), but heart function of Mac-TREM2KO mice was normal prior to MI induction (Supplementary Fig. 4C). Subsequently, both TREM2^flox/flox^ and LysM^Cre^TREM2^flox/flox^ mice underwent LAD ligation surgeries. Supplementary Fig. 4D presents the comparison of serum troponin levels between two groups. Specific knockout of TREM2 in macrophages led to a significant decrease in heart function as EF (23 ± 2.46% versus 10.95 ± 0.63%, *P* < 0.001) and fractional shortening (FS) (10.51 ± 1.20% versus 4.86 ± 0.28%, *P* < 0.001), combined with an increase in LVEDV (LV end-diastolic volume), LVESV (LV end-systolic volume), and so on compared to the control group following MI (Fig. 2A, B and Supplementary Fig. 4E). Furthermore, Masson’s trichrome staining revealed reduced fibrosis in a series of sections over the entire heart, and a significant decrease in left ventricular wall thickness in the infarct region in the Mac-TREM2KO group (Fig. 2C, D and Supplementary Fig. 4E, F).

Next, we conducted a histological evaluation of dead cell elimination, angiogenesis, and fibrosis generation in the post-MI hearts. ACs (apoptotic cardiomyocytes) were significantly increased in Mac-TREM2KO mice compared to the control (Fig. 2E, G). Furthermore, immunofluorescence staining of fibrotic markers, such as Vimentin, Col1a1, or α-SMA along with the proliferation marker Ki67, demonstrated less fibrosis generation in Mac-TREM2KO group (Fig. 2F, H, I and Supplementary Fig. 5A–D). These findings suggested impaired clearance of necrotic cells and reduced fibrosis generation in Mac-TREM2KO mice. However, no significant difference was observed in angiogenesis between the two groups (Fig. 2J and Supplementary Fig. 5E).

Additionally, we performed a cytological assessment of macrophage subsets. We found that TREM2-deficient mice in the MI group exhibited significantly reduced levels of MHCII^low^ macrophages at 7 days post-infarct (Fig. 2K, L and Supplementary Fig. 5F). These findings suggest that TREM2 may regulate macrophage function and polarization after MI. However, TREM2 deficiency did not influence the total number of neutrophils or monocytes in peripheral blood (Supplementary Fig. 5G).

To further confirm which origin of macrophages exert main effect in MI, we blocked the recruitment of circulatory monocytes with a CCR2 chemokine receptor antagonist RS504393 [16], which did not affect the resident macrophage populations. Interestingly, the CCR2 inhibitor resulted in a reduction in EF and an increase in infarct size in the control group, but it did not affect the post-MI cardiac function of Mac-TREM2KO mice (Fig. 2M, N and Supplementary Fig. 5H). This suggested that recruited CCR2^+^TREM2^+^ macrophages played a role in MI, rather than TREM2^+^ resident macrophages.

### Impaired efferocytosis in TREM2-deficient Macrophages

**Fig. 3:**
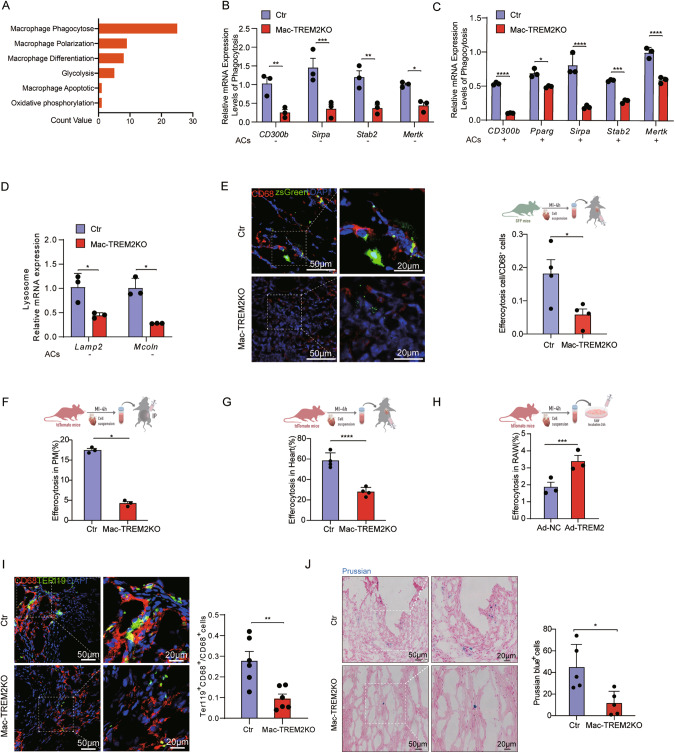
TREM2 deficiency impaired the ability of efferocytosis in macrophages. **A** Gene set enrichment analysis of GEO public dataset (GSE98563). **B** mRNA expression levels of phagocytosis-related genes were examined by qPCR in BMDMs harvested from control and Mac-TREM2KO mice without co-cultivation of apoptotic cardiomyocytes (ACs) (*n* = 3). **C** mRNA expression levels of phagocytosis-related genes were examined by qPCR in BMDMs harvested from control and Mac-TREM2KO mice with co-cultivation of ACs (*n* = 3). **D** mRNA expression levels of lysosome-related genes were examined by qPCR in BMDMs harvested from control and Mac-TREM2KO mice without co-cultivation of ACs (*n* = 3). **E** Representative images and related quantification of immunofluorescence staining of CD68 in hearts of control and Mac-TREM2KO mice administered with ZsGreen^+^ ACs (*n* = 4). Flow cytometric analysis of macrophages phagocytosed Td^+^ ACs in peritoneal macrophages (**F**) and hearts (**G**) of control and Mac-TREMKO mice, and in RAW264.7 treated with adenovirus carrying TREM2 (Ad-TREM2) or negative control (Ad-NC) (**H**) (*n* = 3–4). **I** Representative images and related quantification of immunofluorescence staining results of CD68 and TER119 in heart of control and Mac-TREM2KO mice post-MI (*n* = 6). **J** Representative images and related quantification of Prussian blue staining assay for the phagocytosis of erythrocyte in macrophages in hearts of control or Mac-TREM2KO mice (*n* = 5). Data were expressed as mean ± SEM. Data in **B**–**D** were analyzed by 2-way ANOVA followed by Bonferroni post hoc analysis. Other data were analyzed by Mann–Whitney U tests. ns indicates not significant. *P < 0.05. **P < 0.01. ***P < 0.001.

Macrophages are known for their role as phagocytes responsible for the clearance of necrotic cells. Through bioinformatics analysis (GSE98563), we observed that TREM2 macrophage was primarily associated with enhanced phagocytosis (Fig. 3A). Further, we found that expression of phagocyte and lysosome-associated genes were significantly lower in TREM2KO macrophages but higher in TREM2-overexpressing macrophages (Fig. 3B–D and Supplementary Fig. 6A). Moreover, by incubating Red Zymosan Bioparticles with macrophages, we confirmed TREM2KO macrophages exhibited reduced phagocytosis (Supplementary Fig. 6B), while TREM2-overexpressing macrophages increased phagocytosis (Supplementary Fig. 6C). The efficacy of TREM2 adenovirus-mediated overexpression was confirmed in Supplementary Fig. 6D.

To assess the efferocytosis ability of TREM2^+^ macrophages in the context of ischemic cardiac disease, macrophages were fed with ACs labeled with Td^+^ or Zs^+^ obtained from ischemic hearts of Tdtomato^+^ or ZsGreen^+^ mice after LAD ligation. In an in vivo system, Td^+^ or Zs^+^ ACs were injected into the abdominal cavity and myocardium of control and Mac-TREM2KO mice. In vitro, we incubated Td^+^ ACs with RAW264.7 cells overexpressing TREM2. As expected, both result of in vivo and in vitro experiments supported the hypothesis that TREM2 promoted macrophage efferocytosis (Fig. 3E–H). We further evaluated the ratio of red-blood-cell-engulfing macrophages within the heart to mimic the phagocytosis of cardiomyocytes by macrophages after MI. A significantly lower number of Ter-119^+^CD68^+^ macrophages and less Prussian blue staining further confirmed the defective phagocytosis of Trem2-deficient macrophages on day 7 post-MI (Fig. 3I, J).

### TREM2^+^ macrophages downregulated SLC25A53 expression after efferocytosis through SYK-SMAD4 signaling pathway

**Fig. 4:**
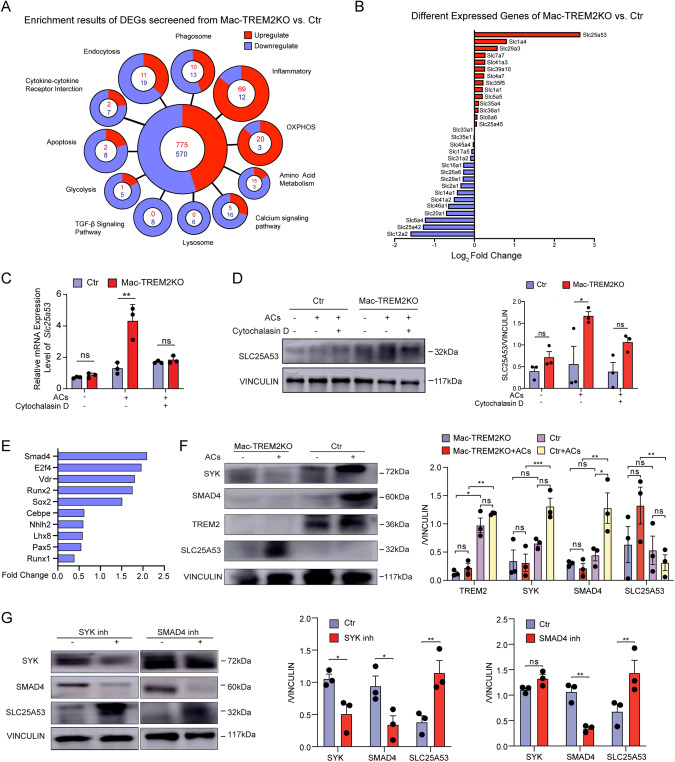
TREM2^+^ macrophages downregulated SLC25A53 expression through SYK-SMAD4-SLC25A53 pathway after efferocytosis. **A** RNA-seq was performed on CCR2^EGFP+^ cells sorted from control and CCR2^cre-EGFP^ TREM2^flox/flox^ mice on day 7 post-MI. Enrichment results of DEGs was shown between these two groups. **B** SLC genes of CCR2^EGFP^^+^ cells that were differentially regulated on day 7 post-MI between control and CCR2^cre-EGFP^ TREM2^flox/flox^ mice were ranked by Log_2_ Fold Change. **C** qPCR was performed to evaluate the mRNA expression levels of SLC25A53 in control and TREM2KO BMDMs stimulated with or without ACs or cytochalasin D. **D** Western blot analysis of SLC25A53 in control and TREM2KO BMDMs stimulated with or without ACs or cytochalasin D. **E** Predicted transcription factors (TFs) that regulated the SLC25A53 were showed, and the screened TFs were ranked as the absolute log_2_ fold change value of bulk RNA-sequencing results. **F** Western blot analysis of SYK, SMAD4, TREM2 and SLC25A53 in control and TREM2KO BMDMs stimulated with or without ACs. **G** Western blot analysis of SYK, SMAD4 and SLC25A53 in control BMDMs treated with SYK inhibitor or SMAD4 inhibitor. *N* = 3 per group. Data were expressed as mean ± SEM. Data in **F** was analyzed by multi-way ANOVA. Other data were analyzed by 2-way ANOVA followed by Bonferroni post hoc analysis. ns indicates not significant. *P < 0.05. **P < 0.01. ***P < 0.001.

To gain insights into the underlying mechanism of how TREM2 affects macrophage functions, we performed RNA-seq analysis on CCR2^+^ cells derived from the infarcted myocardium of CCR2^cre-EGFP^ or CCR2^cre-EGFP^ TREM2^flox/flox^ mice on day 7 post-MI. (the volcano plot and heatmap were shown in Supplementary Fig. 7A, B). Upon MI challenge, TREM2KO macrophages underwent several transcriptional program changes associated with phagocyted-related processes, carbohydrate metabolism (Fig. 4A). In addition, solute carrier 25 member 53 (SLC25A53) was identified as the fourth-ranked DEG (adjusted *P* value < 0.05) after TREM2KO macrophage in vitro (GSE98563) (Supplementary Fig. 7C), and the top-ranking gene in SLC family associated with efferocytosis and metabolism in vivo (Fig. 4B). The first three ranking genes in vitro (GSE98563) showed no significant difference (Supplementary Fig. 7D).

SLCs are membrane proteins that transport diverse molecules located in the plasma or mitochondrial membranes [17, 18]. SLC program is activated following efferocytosis suggests a shift of metabolism program and metabolites changed in this shift can influence substantial cells in the microenvironment [17]. The expression of SLC25A53 increased upon stimulation of ACs in TREM2KO BMDMs, whereas it decreased in the presence of cytochalasin D, a phagocytic inhibitor, suggesting that the process of efferocytosis affected the expression of SLC25A53 in TREM2 macrophage (Fig. 4C, D). In order to explore how TREM2 regulated SLC25A53, we used KnockTF to screen the potential regulator. The result suggested that SMAD4 was the most critical regulator which functioned as a down-regulator of SLC25A53 (Fig. 4E), and TREM2 might promote SMAD4 expression through activation of SYK. We verified this hypothesis between control and TREM2KO macrophages (Fig. 4F). The use of SYK inhibitor or SMAD4 inhibitor was able to rescue the phenotype, demonstrating the SYK-SMAD4-SLC25A53 signaling pathway (Fig. 4G). Collectively, we concluded that TREM2 inhibited the expression of SLC25A53 through SYK-SMAD4-SLC25A53 pathway after efferocytosis.

### TREM2^+^ macrophages affect NAD^+^ transporting in mitochondria through SLC25A53 after efferocytosis

**Fig. 5:**
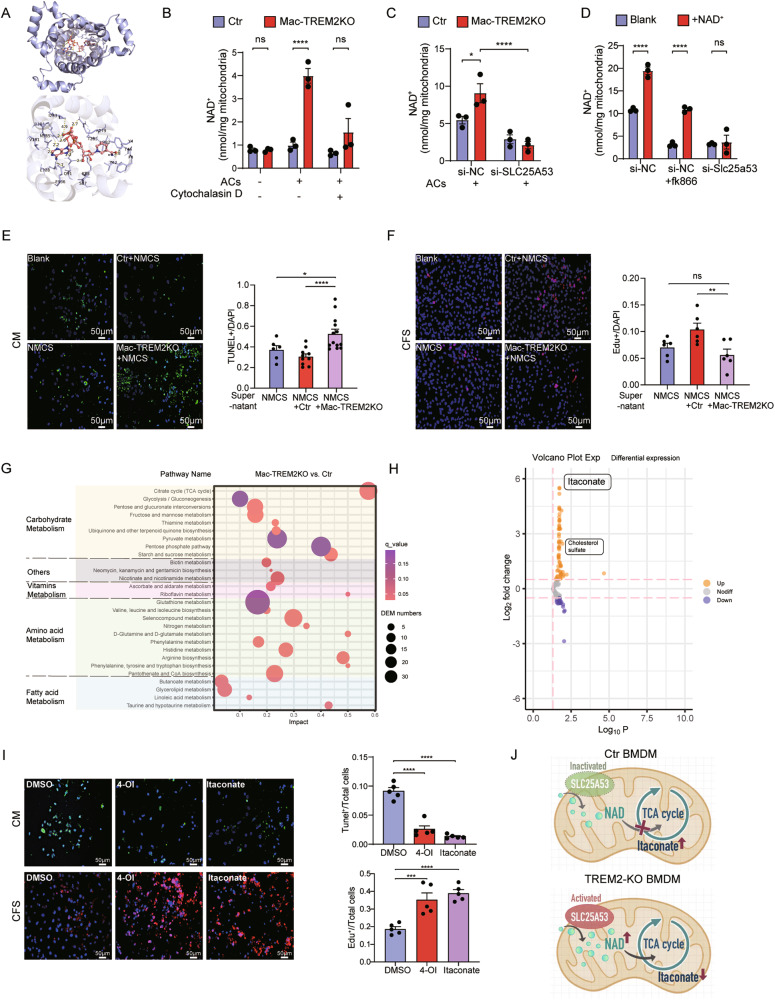
TREM2^+^ macrophages affect NAD^+^ transporting in mitochondria through SLC25A53 after efferocytosis, followed by promoting the release of itaconate. **A** Predicted docking module of SLC25A53 and NAD^+^ was analyzed by Autodock and exhibited with PyMol. **B** NAD^+^ content was measured in isolated mitochondria of control and TREM2KO BMDMs treated with or without the stimulation of ACs and cytochalasin D (*n* = 3). **C** NAD^+^ content was measured in isolated mitochondria of control and TREM2KO BMDMs with or without knockdown of SLC25A53 after co-cultivation with ACs (*n* = 3). **D**, NAD^+^ content was measured in isolated mitochondria of control BMDMs with or without knockdown of SLC25A53 and further application of FK866 and exogenous NAD^+^ (*n* = 3). Representative immunofluorescence images and related quantification of TUNEL^+^ cardiomyocytes (CM) (**E**) and Edu^+^ cardiac fibroblasts (CFS) (**F**) after treatment with supernatants from control or TREM2KO BMDMs that was preincubated with necrotic myocardial cell supernatants (NMCS) (*n* = 6-13). **G** Results of LC–MS profile and enrichment results of the metabolic pathway in control and TREM2KO BMDMs after being stimulated with ACs. **H** Volcano plot of significant metabolites of mitochondria in control vs TREM2KO BMDMs after being stimulated with ACs. **I** Representative immunofluorescence images and related quantification of EDU^+^ CFS and TUNEL^+^ CM with stimulation of DMSO, 4-octyl itaconate (4-OI) or itaconate (*n* = 5). **J** Scheme of SLC25A53 transporting NAD^+^ into mitochondria and further influencing TCA cycle and itaconate production. Data were expressed as mean ± SEM. Data in **B**–**D** were analyzed by 2-way ANOVA followed by Bonferroni post hoc analysis. Other data were analyzed by Mann–Whitney U tests. ns indicates not significant. *P < 0.05. **P < 0.01. ***P < 0.001.

The results drove us to investigate the function of SLC25A53, which had not been fully revealed. An important paralog of this gene was SLC25A51, a mammalian transporter capable of importing NAD^+^ into mitochondria [19]. We suspected that SLC25A53 could also transport NAD^+^. To explore this hypothesis, we employed AutoDock Vina to simulate molecular docking between SLC25A53 and NAD^+^, and the binding energy was -8 kcal/mol, indicating a strong potential for real binding (Fig. 5A). Then, we assessed the ability of SLC25A53 to transport NAD^+^ in vitro. There was a significant increase in mitochondrial NAD^+^ levels after efferocytosis in TREM2KO, but the increase disappeared when efferocytosis was blocked by cytochalasin D (Fig. 5B). Moreover, the elevated level of NAD^+^ in mitochondria of TREM2KO was inhibited after transfection with si-SLC25A53 (Fig. 5C). Knock down efficiency was certificated in Supplementary Fig. 8A, B. Without NAD^+^ addition, the decreased NAD^+^ level of mitochondria in si-SLC25A53 group was almost equivalent to control treated with NAD^+^ synthesis inhibitor, FK866, while exogenous NAD^+^ did not increase the mitochondria NAD^+^ content in si-SLC25A53 (Fig. 5D). Moreover, the deficiency of TREM2 in macrophages as well as the knockout of SLC25A53, did not influence the total cellular NAD^+^ levels (Supplementary Fig. 8C, D). These suggested that SLC25A53 play a role in the transport of NAD^+^ into mitochondria in TREM2^+^ macrophages while not affecting the overall cellular levels of NAD^+^.

Additionally, knockout of TREM2 or knockdown of SLC25A53 did not influence the expression of SLC25A51 (Supplementary Fig. 8E, F). Knockdown of SLC25A51 had less influence in the process of mitochondria NAD^+^ uptake (Supplementary Fig. 8G), indicating that SLC25A53 was the main NAD^+^ transporter rather than SLC25A51 in TREM2^+^ macrophages. Furthermore, we found that SLC25A53 deficiency did not affect efferocytosis (Supplementary Fig. 8H).

### LC–MS results revealed that TREM2 promoted the release of itaconate in macrophage via SLC25A53 and metabolic change after efferocytosis

Research has shown that metabolism is reprogrammed after efferocytosis and metabolic by-products of efferocytosis can influence surrounding cells [17]. Combined with the decrease of ACs and increased fibrosis in Mac-TREM2KO after MI, we aimed to investigate whether TREM2^+^ macrophages could secrete products that influenced cardiomyocytes or fibroblasts within the ischemic microenvironment. We used the supernatants of necrotic cardiomyocytes to stimulate control and Mac-TREM2KO BMDMs, then the conditioned supernatants from these BMDMs were collected and utilized to stimulate ischemic and hypoxic cardiomyocytes as well as fibroblasts. Remarkably, we observed increased cardiomyocyte apoptosis and reduced fibroblast proliferation when stimulated with supernatants derived from TREM2KO macrophages (Fig. 5E, F). In contrast, when TREM2 was overexpressed in BMDMs, the dynamics following injury skewed towards enhanced fibroblast proliferation and reduced cardiomyocyte apoptosis (Supplementary Fig. 9A). Based on the results and previous research [17], we formulated a hypothesis that TREM2^+^ macrophages downregulate SLC25A53 expression after efferocytosis, thus influencing glycometabolism, whose metabolic byproducts might have an impact on cardiomyocytes and fibroblasts. To test the conjecture and search for possible metabolites, we incubated BMDMs with apoptotic H9C2 cells and then performed LC–MS metabolic profiling on isolated mitochondria. We observed higher levels of TCA cycle metabolites in the TREM2KO BMDMs (Fig. 5G), and itaconate was the most significant one among the metabolites analyzed (Fig. 5H). We further verified in SLC25A53 deletion groups in which itaconate also showed increased levels (Supplementary Fig. 9B). As itaconate derivative 4-OI (4-octyl itaconate) has been found to more closely mimic endogenous itaconate [20, 21], we compared the effect of 4-OI and itaconate on ischemic and hypoxic cardiomyocytes or fibroblasts. Our results demonstrated both 4-OI and itaconate-treated groups exhibited a higher proliferation ratio of fibroblasts (Fig. 5I and Supplementary Fig. 9C) and a lower apoptosis ratio of cardiomyocytes compared to the DMSO-treated cells (Fig. 5I). Action potential (AP) detection further demonstrated that 4-OI can help decrease AP duration and improve the contractility of cardiomyocytes (Supplementary Fig. 9D). We also established hiPSCs-derived cardiomyocytes to confirm the rescue effect of 4-OI on cardiomyocytes (Supplementary Fig. 9E).

Itaconate is derived from cis-aconitate in the TCA cycle in macrophages [22]. This organic acid is thought to be the result of a potential breakpoint of the conversion of isocitrate (ICIT) to oxoglutarate (AKG) in the metabolic flow [23]. Furthermore, the disturbance of NAD^+^ transport into mitochondria is followed by TCA cycle interruption, and we found that this disturbance coincides with the breakpoint that leads to the production of itaconate. This suggested that SLC25A53 downregulation in TREM2^+^ macrophage results in the upregulation of itaconate by interrupting TCA cycle through inactive NAD^+^ transport. (Fig. 5J). Research shows that this breakpoint effect was accompanied by significant transcriptional downregulation of IDH1, the enzyme that interconverts ICIT-to-AKG [23]. However, our result indicated that the observed breakpoint was mainly dependent on the defective import of NAD^+^ into mitochondria in TREM2^+^ macrophages caused by the downregulation of SLC25A53, rather than transcriptional differences in IDH1 (Supplementary Fig. 9E, F).

### Improvement of left ventricular remodeling after injection of TREM2 adenovirus

**Fig. 6:**
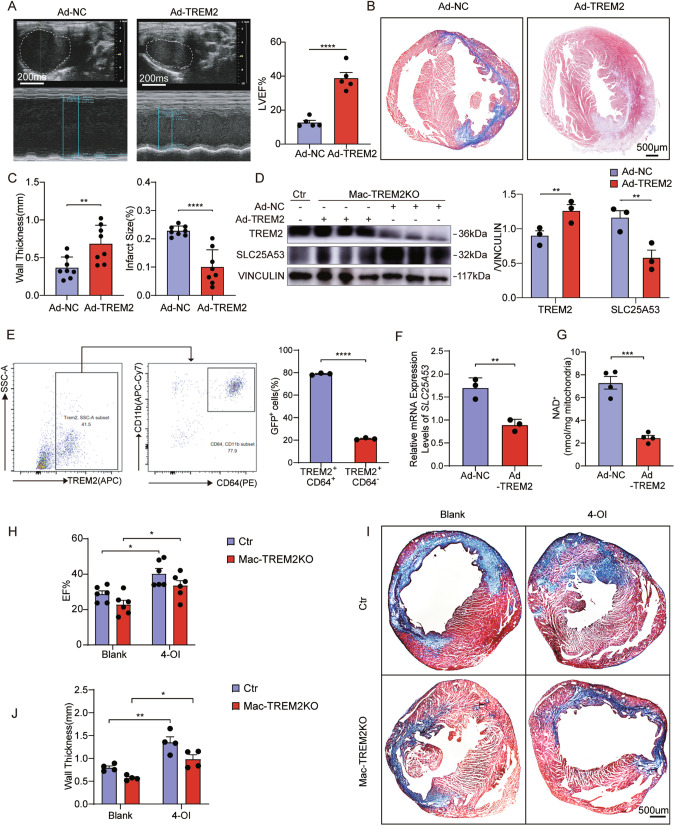
Improvement of left ventricular remodeling after in vivo injection of TREM2-overexpressing adenovirus. **A** Representative M-mode echocardiogram images and analysis of EF in Mac-TREM2KO mice intramyocardially administered with Ad-NC or Ad-TREM2 on day 7 post-MI (*n* = 5). **B** Representative images of Masson’s trichrome staining of heart tissues (*n* = 5). **C** Quantification of left ventricular wall thickness and infarct size of groups described in B (n = 8). **D**, Western blot analysis of TREM2 and SLC25A53 in heart of control mice and groups described in (**A**) (*n* = 3). **E** Flow cytometric analysis of percentage of GFP^+^ cells in TREM2^+^ CD64^+^ cells and TREM2^+^ CD64^-^ cells in heart of Mac-TREM2KO mice administered with Ad-TREM2 (*n* = 3). **F** Relative SLC25A53 mRNA expression level in macrophages sorted from heart of groups described in **A** (*n* = 3). **G**, NAD^+^ content in isolated mitochondria of macrophages sorted from heart of groups described in **A** (*n* = 4). **H** Echocardiographic analysis of EF in control and Mac-TREM2KO mice administered with blank or 4-OI on day 7 post-MI. 50 mg/kg 4-OI was intraperitoneally injected every 48 h, beginning immediately after the MI surgery (*n* = 6). **I**, **J** Representative images of Masson’s trichrome staining and quantification of wall thickness and fibrosis area of heart tissue of groups described in **I** (*n* = 4). Data were expressed as mean ± SEM. Data in **D** and **H**–**J** were analyzed by 2-way ANOVA followed by Bonferroni post hoc analysis. Other data were analyzed by Mann–Whitney U tests. ns indicates not significant. *P < 0.05. **P < 0.01. ***P < 0.001.

We performed the rescue experiment by injecting adenovirus carrying TREM2 (Ad-TREM2) and control (Ad-NC) into myocardial immediately after LAD ligations to verify the effects of TREM2 in vivo. Mice treated with Ad-TREM2 had significantly higher EF% on day 7 post-MI (approximately 20% improvement compared to control group) (Fig. 6A and Supplementary Fig. 10A). Histologically, Ad-TREM2 group exhibited better remodeling of LV (Fig. 6B, C and Supplementary Fig. 10B), with higher expression of TREM2 and lower expression of SLC25A53 (Fig. 6D). Supplementary Fig. 10C showed fibrosis was no difference in sham mice treated with Ad-NC and Ad-TREM2. Nevertheless, there was no significant difference in mortality among the different groups (Supplementary Fig. 10D). The serum troponin I levels were shown in Supplementary Fig. 10E. Moreover, it showed nearly 80% of Ad-TREM2 (GFP^+^TREM2^+^) cells in the heart were CD64^+^ macrophages (Fig. 6E). Furthermore, cardiac macrophages sorted from Ad-TREM2 mice had a lower level of SLC25A53 expression and reduced NAD^+^ content (Fig. 6F, G). These results provided compelling evidence that TREM2 overexpression downregulated SLC25A53 in macrophages and promoted both functional and structural improvements of infarcted hearts in vivo.

We further investigated the inflammatory level in vivo and in vitro. As expected, TREM2 deletion decreased the expression of anti-inflammatory factors and increased pro-inflammatory factors, while TREM2 overexpression yielded the opposite outcome (Supplementary Fig. 11A, B). We also investigated the reparative effects of itaconate on cardiac injury following MI. It was shown that 4-OI injection led to a significant recovery in EF% (Fig. 6H and Supplementary Fig. 11C). Masson trichrome staining showed an increase of wall thickness in mice administered with 4-OI (Fig. 6I, J). These results suggested the potential treatment effect of itaconate derivatives in myocardial infarction.

## Discussion

Our study has revealed the important role of TREM2 in orchestrating macrophage efferocytosis, and subsequently modulating NAD^+^ transport by suppressing the expression of SLC25A53, a mitochondrial carrier for NAD^+^ in macrophages. Furthermore, we have demonstrated that TREM2 augments the production of itaconate by interrupting the TCA cycle after efferocytosis. Collectively, these findings highlight the significance of TREM2 as a critical regulator of the efferocytosis-TCA cycle-immune metabolites axis in macrophages, suggesting its potential as a promising therapeutic target for restoring cardiac function after myocardial infarction.

The myeloid receptor, TREM2, has garnered scientific attention due to its association with the risk of Alzheimer’s disease [24], as well as its roles in chronic diseases as a pathology-induced immune signaling pathway in macrophages [8], such as promoting phagocytosis, maintaining metabolic homeostasis and preserving cell survival [25]. Our study adds to the understanding of TREM2’s function in acute disease by demonstrating its crucial role in preserving post-MI cardiac function. Specifically, we found that knockout of TREM2 in myeloid lineages in mice led to a 10% decrease in EF, whereas overexpression of TREM2 rescued EF by 20%. Macrophages are believed to be the key regulator of the immune system, providing a protective response in resolving local damage caused by sterile inflammation in the heart [26]. Several studies have emphasized the importance of targeting post-MI macrophages in cardiac remodeling to achieve long-term benefits in animal models [27]. Our results demonstrate that TREM2 acts through macrophages after MI, which expression in the heart increases on day 3 and peaks around day 7 post-MI. This timeframe aligns with the macrophage-mediated repair process post-MI, characterized by the clearance of debris, release of anti-inflammatories, and metabolic reprogramming [28].

TREM2 is primarily recognized as a phagocytic receptor, as identified by N’Diaye et al. [29], while efferocytosis is essential for promoting cardiac repair [30]. Thus, we initially investigated this process and found that the primary mechanism by which TREM2 prevents post-MI cardiac remodeling and preserves cardiac function is through efferocytosis. Efferocytosis involves not only the clearance of cell debris but also the hydrolysis of apoptotic cells, production of abundant metabolites, regulation of cell metabolism and function, and activation of macrophage proliferation [31]. Ultimately, efferocytosis results in the attenuation of inflammation and restoration of tissue homeostasis [32]. Our bioinformatic analysis revealed that phagocytosis, inflammation, and OXPHOS/glycolytic were the top three significantly altered pathways in CCR2^cre-EGFP^TREM2^flox/flox^ mice. Additionally, our findings indicate that TREM2^+^ macrophages present anti-inflammatory function.

Following efferocytosis, the solute carrier family (SLC) program is activated, and the metabolic byproducts can influence other cells in the microenvironment [17]. SLC25, a subfamily of SLC, is a large family of mitochondrial inner membrane transporters involved in various metabolic pathways and cell functions. While most members of this family function as strict counter-exchangers of chemically related substrates, the functions of many SLC members remain unknown [18, 19, 33, 34]. SLC25A53, which is highly expressed in various brain cells, including neurons, microglial cells, lymphocytes, and macrophages in the bone marrow (the Human Protein Atlas), is one of the most outstanding transcriptional landscape changes in TREM2KO macrophages, but its function remains unclear. Our study reveals that SLC25A53, an isoform of SLC25A51, is the primary NAD^+^ transporter in macrophages, whereas the expression of SLC25A51 in macrophages is minimal and has no significant role in our data, suggesting that SLC25A51 may play a more prominent role in parenchymal cells.

**Fig. 7:**
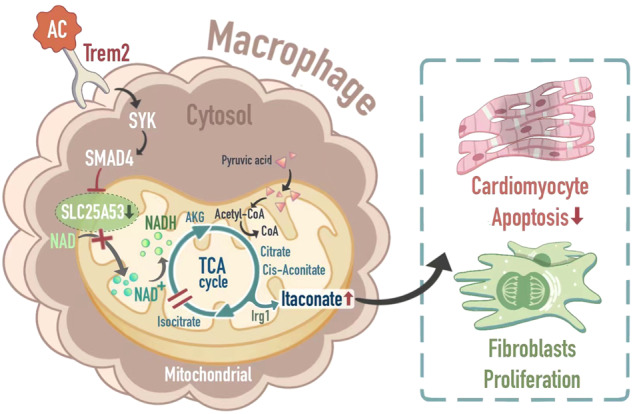
Mechanism diagram: TREM2 Promote Cardiac Repair in Myocardial Infarction via SLC25A53 and Carbohydrate Metabolism after Improving Efferocytosis of Macrophage. When TREM2 sensed apoptotic cells or damaged tissues and drove efferocytosis process, the TREM2-SYK-SMAD4 signaling pathway was activated, which inhibited the transcription of SLC25A53. The low expression of SLC25A53 resulted in low efficiency of NAD^+^ transporting thus presenting breakpoint in TCA. Thus, anti-inflammatory organic acids such as itaconate, were released in larger amounts and resulted in higher efficiency of post-MI myocardial repair.

Indeed, macrophage function in tissue repair and resolution is influenced not only by signaling pathways but also intracellular metabolic pathways [35]. Our study is the first to report the upregulation of SLC25A53 following efferocytosis and its impact on TCA cycle in TREM2KO macrophages. In the presence of TREM2, SLC25A53 expression is decreased, resulting in a decrease in mitochondrial NAD^+^ levels and a potential breakpoint in the metabolic flow at the isocitrate-to-α-ketoglutarate conversion in the TCA cycle. As a result, there is a downregulation of TCA cycle and subsequent OXPHOS. However, pyruvate can still enter the TCA cycle. This leads to the accumulation of upstream cis-aconitate and increased itaconate production, which promotes tissue repair after myocardial infarction (MI) (Fig. 7).

In fact, various natural metabolites are generated through glycolysis and the TCA cycle. In our study, we observed that impaired efferocytosis in bone marrow-derived macrophages (BMDMs) from LysM^Cre^TREM2^flox/flox^ mice has significant impact on itaconate production. Itaconate has been reported to promote the establishment of an anti-inflammatory tissue environment [36] and inhibit TET DNA dioxygenases, thus attenuating inflammatory responses [37]. Furthermore, itaconate and its derivatives suppress the inflammatory response and change the polarization of macrophages through the JAK1 pathway [38]. Notably, we found that itaconate can inhibit the apoptosis of cardiomyocytes and stimulate the proliferation of fibroblasts. Itaconate has been proven to have anti-inflammatory function, and dimethyl itaconate (DI), a derivative of itaconate, has exhibited treatment effects in myocardial ischemia-reperfusion injury [39]. This provides another example of how immune metabolic by-product resulting from efferocytosis can influence neighboring cells and their functions.

This study delivers a mouse centered mechanistic assessment of the importance of TREM2 in myocardial repair. A TREM2-based intervention was tested in mice. Moreover, TREM2 agonists is undergoing phase III clinical trials in Alzheimer’s disease. Whether it has the same rescue effect in MI patients need to be further investigated. In this context it is also important to point out that all mouse experiments were performed in a model of permanent LAD ligation rather than LAD ischemia-reperfusion. Finally, timing of the TREM2 delivery is at the essence as the balance of inflammation and repair is tightly controlled. Hence, further studies on timing, dosage, and tissue-specific delivery are warranted.

In summary, our study provides novel insights into the role of TREM2 macrophages in MI and sheds light on the underlying mechanisms. Our findings open new possibilities for enhancing cardiac repair after injury. The identification of TREM2 macrophages as a promising therapeutic target for the treatment of MI holds great potential in the field of clinical research. We anticipate that our results will have significant implications for further investigations and the development of innovative treatments in this important clinical domain.

## Methods

### Patients and clinical specimens

Written informed consent was obtained from patients involved in the study under the supervision of the local Ethical Committee of Shanghai Tenth People’s Hospital. Throughout the study, the guidelines of the Declaration of Helsinki were observed. 10 healthy volunteers and 29 patients who survived ST-segment elevation MI participated in our study from January 2023 to April 2023. Blood samples from MI patients were collected at day 1 and day 5 respectively. MI was defined according to the *Guideline for the Management of ST-Elevation Myocardial Infarction* [40]. Detailed medical and family history of the healthy volunteers was obtained, while none had documented cardiovascular disease, severe kidney or liver disease, or malignancy. All participants’ blood samples were collected after an overnight fast following hospital admission. Basic characteristics were shown in Supplementary Table 1. The PBMCs were extracted using the EasySep™ Direct Human PBMC Isolation Kit (19654, STEMCELL Technologies) according to the manufacturer’s instructions. Information on TREM2’s primer was presented in the Supplementary Table.

### Mouse models

All animal procedures were approved by the Animal Care and Use Committees of Shanghai Tenth People’s Hospital affiliated with Tongji University for animal welfare. Experiments were conducted in compliance with the Guide for the Care and Use of Laboratory Animals published by the National Institutes of Health (NIH Publication, 8th Edition, 2011). TREM2^flox/flox^ mice (purchased from Shanghai Model Organisms) were crossbred with LysM^Cre^ mice (purchased from Nan Jing Xietong Biotechnology company) to generate LysM^Cre^TREM2^flox/flox^ (termed Mac-TREM2KO) mice [19]. TREM2^flox/flox^ littermates served as controls (Ctr). All mice were maintained in the C57BL/6 background, and 8 weeks-old male mice were used in this study. C57BL/6-Ccr2em1^(cre/ERT2/EGFP)^/J (CCR2^cre-EGFP^) mice, donated by Professor Bo Peng from the Medical School of Fudan University, were crossed with TREM2^flox/flox^ mice for the production of fluorescent macrophages. Genotyping of all mice was performed using PCR. CCR2 chemokine receptor antagonist RS504393 (ab120183, Abcam) was intraperitoneally injected at a dose of 2 mg/kg/d from day 1 to day 6 post-MI.

### Murine MI model and echocardiography

Mice were anesthetized with pentobarbital sodium (50 mg/kg, Intraperitoneal injection) and intubated with a 22-gauge incubation tube while being mechanically ventilated with a small rodent respirator. A left thoracotomy was performed between the third and fourth intercostal space. The left anterior descending coronary artery (LAD) segment corresponding to approximately 1.5 mm to 2 mm below the tip of the left auricle was permanently ligated with an 8-0 monofilament nylon suture. Then, the chest wall was closed, and air in the thorax was evacuated. Mice in the sham group underwent the same procedure without LAD ligation. After surgery, cardiac function was examined by echocardiography. All mice were carefully monitored each day after MI. Mice that died immediately or had an ejection fraction (EF) > 50% after surgery were excluded from this study. Heart samples were harvested at different time points for subsequent experiments. Transthoracic 2-dimensional and M-mode images were obtained using Vevo 2100 system with a 20-MHz transducer (Vevo® 2100 system, Fujifilm Visual Sonics). Animals were killed by cervical dislocation under isoflurane anesthesia (induced by isoflurane inhalation 4.0%).

### Immunofluorescence staining and laser confocal fluorescence microscopy analysis

Immunofluorescent staining experiments were performed on 8 μm cardiac cryostat sections obtained from sham or MI mice as described previously [41]. Cells were seeded at 1 × 10^5^ cells/well on 6-well culture plates. The sections and cells were fixed with freshly prepared 4% paraformaldehyde for 10 min, followed by permeabilization with 0.2% Triton X-100 in PBS for 8 min. Cryosections and cells were incubated with anti-TREM2 (AF1729, R&D), anti-CD68 (NB100-683, Novus), anti-MAC2 (H2120, Santa Cruz), anti-Ly6G (127601, Biolegend), anti-CD11c (sc-376764, Santa Cruz), anti-α-SMA (ab32575, Abcam), anti-Actin (A2172, Sigma), anti-Vimentin (GB12192, Servicebio), anti-CD31 (GB13063, Servicebio), anti-Col1a1 (SC-8784, Santa Cruz), and anti-Ki67 (AF7649, R&D) overnight at 4 °C. Normal isotype IgG (sc-2025, Santa Cruz) was used as TREM2 negative control. After washing with phosphate-buffered saline (PBS), secondary antibodies (Alexa Fluor 488-conjugated donkey anti-sheep, Alexa Fluor 647-conjugated donkey anti-sheep, Alexa Fluor 488-conjugated goat anti-rabbit, Alexa Fluor 488-conjugated donkey anti-rat, Alexa Fluor 594-conjugated goat anti-rat, Alexa Fluor 594-conjugated donkey anti-rabbit, and Alexa Fluor 594-conjugated goat anti-mouse; Invitrogen) were added for a 1-h incubation at 37 °C in the dark. Apoptotic cardiomyocytes (ACs) in infarcted myocardium were assessed using TUNEL assay (40307ES20, YEASEN). Cell proliferation was assessed using EDU assay (40276ES60, YEASEN). The number of EDU-positive cells and TUNEL-positive cells was counted in 6-8 randomly selected fields of each sample under ×20 magnification. Nuclei were labeled with DAPI (Vector Laboratories), and cells were visualized using an LSM710 laser confocal microscope (Carl Zeiss, Germany).

### Histology

Heart samples were fixed by 4% paraformaldehyde, embedded in paraffin, and cut into 4 μm transverse sections at different levels. Masson’s trichrome staining was performed on paraffin-embedded sections at the papillary and apical level and then examined under ordinary polychromatic light or polarized light microscope to determine the extent of cardiac fibrosis. For each section, ten to fifteen images were acquired from randomly selected fields in both infarct and non-infarct areas. Image analysis was conducted using Image J software (V1.49, National Institutes of Health). Serial heart cross-sections cut above the level of ligature as well as 5, 1.0, 1.5, and 2.0 mm distal to the suture were stained with Masson’s trichrome for infarct size evaluation.

For immunohistochemistry staining, the sections were incubated with primary anti-TREM2 (AF1729, R&D), anti-α-SMA (ab32575, Abcam), and anti-Vimentin (GB12192, Servicebio) overnight at 4 °C after blocking the endogenous peroxidase activity. Following the incubation, a Vectastain Elite ABC kit (PK-8502, Vector Laboratories) was used according to the manufacturer’s instructions. After visualization with 3,3’ diaminobenzidine, the sections were counterstained with hematoxylin.

For triphenyltetrazolium chloride (TTC) staining, fresh frozen hearts were cut transversely into 1.2 mm-thick slices and stained with 2% TTC staining solution (G3005, Solarbio) for 20 minutes in a 37 °C water bath, then fixation for 12 hours in 4% neutral buffered formaldehyde.

### Protein extraction and western blot

Whole cells from in vitro experiments were prepared by 1× cell lysis buffer (9803, Cell Signaling Technologies) containing protease inhibitors (04693159001, Roche Molecular Biochemicals). Lysates were cleared by centrifugation.

Protein concentrations were determined using the BCA protein assay. Proteins were separated with SDS–PAGE, transferred to polyvinylidene fluoride membranes, and incubated overnight at 4 °C with primary antibodies, including anti-TREM2 (AF1729, R&D), anti-Vinculin (sc-73614, Santa Cruz), anti-SLC25A53 (STJ193987, ST John’s Laboratory), anti-SYK (ab40781, Abcam), and anti-SMAD4 (ab40759, Abcam). The membranes were then incubated with secondary antibodies for one hour, and bands were visualized using chemiluminescence (TANON-2500, TANON), then viewed under Amersham Imager 600 system (GE Healthcare, USA). Uncropped western blots are provided in Supplementary Material.

### Quantitative PCR

Total RNA samples from the sorted cells, tissues, and whole mouse blood were prepared using RNeasy Mini Kit (Qiagen, Germany) or TRIzol reagent (Thermo Fisher, USA) according to the manufacturer’s instructions, and reverse-transcribed using HiScript III RT SuperMix reverse-transcription reagent kit (7110203, Vazyme Biotech). Quantitative PCR (qPCR) was performed using a Roche Lightcycler 96 (USA). The level of GAPDH and 18 S ribosomal RNA were used as the internal control for mouse experiments. Each reaction was performed in duplicate, and the changes in relative gene expression normalized to the internal control levels were determined using the relative threshold cycle method. Information on primers was presented in the Supplementary Table.

### Flow cytometry

The hearts were dissected, minced with fine scissors, and enzymatically digested in Hanks’ Balanced Salt Solution (HBSS, H9269, Sigma-Aldrich) with a cocktail of 1 mg/mL type II collagenase (1148090, Merck), 100 U/mL hyaluronidase (H4272, Sigma-Aldrich), and 100 U/mL DNase I (69182, Sigma-Aldrich). The mixture was agitated first with gentleMACS Dissociator (130093235, Miltenyi Biotec) and then dissociated on a shaking table concentrator for 40 min at 37 °C. Following the digestion, the tissue samples were triturated and passed through a 70 µm cell strainer (BD Falcon, USA). The obtained cells were centrifuged at 400 rpm at 4 °C for 5 minutes, counted after erythrocyte lysis, and washed with HBB buffer solution (HBSS buffer with 2% fetal bovine serum [FBS] and 0.2% bovine serum albumin [BSA]) for further analysis. The above cell suspensions from mouse hearts were stained with live/dead fixable viability stain 780 (565388, BD Horizon) for 8 min. Cell suspensions were blocked with CD16/32 antibody (101320, Biolegend) for 15 min and then stained with corresponding fluorescently labeled antibodies, anti-CD45 (103108, Biolegend), anti-CD11b (557397, Biolegend), anti-CD64 (139323, Biolegend), anti-Ly6C (128018, Biolegend), anti-CCR2 (150627, Biolegend), anti-MHCII (107631, Biolegend), anti-Ly6G (127641, Biolegend), anti-F4/80 (123131, Biolegend), and anti-TREM2 (FAB17291C and FAB17291R, R&D), afterward diluted in PBS solution at the indicated concentration. The results were presented in percentage or the cell number per microgram of tissue. Flow cytometric analysis and cell sorting were performed on LSRFortesa and FACSAria instruments (BD Biosciences) and analyzed using FlowJo software (V10.0.7, USA).

### Injection of adenovirus in the heart

The injection was performed right after the ligation of LAD. Adenovirus carrying either TREM2 (Ad-TREM2) or negative control (Ad-NC, Ctr) under the promoter of cytomegalovirus were injected into the left ventricular anterior wall (three sites, 10 μL/site without dilution, 2 × 10^10^ PFU) using a micro syringe. Ad-TREM2 and Ad-NC were synthesized by Shanghai Genechem Co., Ltd (China). The sequence of Ad-TREM2 was NM_001272078 (Genebank).

### Generation of bone marrow-derived macrophages (BMDMs)

Bone marrow cells were isolated from the femurs of adult mice and differentiated into BMDMs using 40 ng/ml M-CSF (HY-P7050, MCE) plus 10% FBS and 1% penicillin-streptomycin for 5–7 days. BMDMs (2 × 10^5^ cells/mL) were seeded into 6-well culture plates.

### Primary cardiomyocyte and fibroblast culture

Rat cardiomyocyte cultures were prepared from two-day-old male and female rat pups. The fresh myocardial tissue of the rats was cut into small heart fragments and digested with Collagenase II (9001-12-1, Sigma). After the digestion of tissues 6-7 times, 5 minutes each time in 37 °C in water bath, the digested cells from the tissues were collected except that supernatant was discarded for the first time. The cells were then cultured in DMEM containing 20% FBS in 10 cm dish and kept at 37 °C and 5% CO_2_. After 1 hour, fibroblasts became adherent and cardiomyocytes remained in the supernatant. Cardiomyocytes were then collected separately and cultured in 6-well plates. When the cell density reached 70%-80%, EDU assay (40276ES60, YEASEN) was conducted on fibroblasts and TUNEL assay (40307ES20, YEASEN) was conducted on cardiomyocytes according to manufacturer’s instructions. The ATP content was quantified following the guidelines provided by the ATP assay kit from YEASEN (Shanghai, China) and normalized to the maximum content.

### Cardiomyocyte differentiation from hiPSCs

Human cardiomyocytes derived from hiPSCs (human Induced pluripotent stem cells) in our experiments were developed according to previous studies [42]. hiPSCs were cultured in mTeSR^TM^1 for 3 days, and then administrated by Wnt signaling modulators. On day 17, the generated cardiomyocytes were digested and reseeded on a monolayer. Then, 1640 + B27 + AC + DMEM (no glucose) +lactate was used to culture cells (Reviewer-only Fig. 2). On day 22, Cells were treated with or without 100 μM and 250 μM 4-OI and incubated under 5%O2 with 10% FBS or 2% O2 with 2% FBS separately.

### Measurement of Action potential of primary cardiomyocyte

We used whole-cell patch-clamp recording technique. When the whole-cell seal was formed, the cells were given current stimulation at 5 ms, 500 Pa in the current clamp mode, and the evoked action potential was recorded.

### Exocrine in vitro assay

BMDMs were isolated from Mac-TREM2KO and control mice separately. They were then treated with necrotic myocardial cell supernatants (NMCS) in fresh culture media (1:1) for 24 h. The supernatants (20 μg/ml) of these stimulated BMDMs were collected to further stimulate fibroblasts and ischemic and hypoxic cardiomyocytes for 24 hours (the supernatant-to-medium ratio was 1:2). The medium was changed and the culture continued for 12 hours [43]. For TREM2 overexpressed on WT mice BMDMs, they were cultured in 10% FBS with Ad-NC or Ad-TREM2 adenovirus (GENECHEM) for two consecutive days, then treated with NMCS. NMCS were induced using the freeze-thaw technique [39]. Freshly excised 12-week-old C57BL/6 mouse hearts (5–8 hearts/batch) were rinsed in cold PBS to remove red-blood cells and then minced into approximately 10 pieces in chilled PBS. In order to induce apoptosis in myocardial cells, the minced tissue was resuspended in PBS and placed in a conical tube that was subjected to 3 freeze-thaw cycles (10 min in methanol on dry ice [<-80 °C] and 3 minutes in a water bath at 37 °C). NMCS were centrifuged at 12000 × *g* for 10 min to separate the pellet (cell fraction and nuclear fraction) from the cytoplasm (supernatant). The protein content of the supernatant was determined using the BCA assay (Pierce, Thermo Scientific).

### Itaconate stimulation

Fibroblasts and cardiomyocytes were isolated from neonatal rats’ hearts. They were treated respectively with 100 μM 4-octyl itaconate (4-OI) and 100 μM itaconate. Cardiomyocytes were cultured in low-serum (2%) DMEM at 37 °C, 93% N_2_, 5% CO_2_, and 2% O_2_ for 24 hours. Fibroblasts were cultured at 37 °C in a humidified atmosphere containing 5% CO_2_.

### Efferocytosis experiments in vivo and in vitro

Experimental MI was conducted in ZsGreen or Tdtomato transgenic mice. The apoptotic myocardial cells were extracted from ZsGreen or Tdtomato transgenic mice (Td^+^ ACs or Zs^+^ ACs) and then injected into the hearts and abdominal cavities 4 h after MI, respectively. In vitro experiments, the apoptotic cells (ACs) were harvested from H9C2 cell lines (purchased from Shanghai Zhongqiaoxinzhou Biotech, China) and treated with ultraviolet radiation for 2 hours. ACs were co-cultured with RAW264.7 (purchased from Shanghai Zhongqiaoxinzhou Biotech, China).

### NAD^+^/NADH assays

After BMDMs were cultured for 5 days, knockdown of SLC25A53 expression was performed using target-specific SLC25A53 siRNA or SLC25A51 siRNA (GenePharma, China), with negative siRNAs (GenePharma, China) as control. Cells were transfected with jetPrime Transfection Reagent (Polyplus) according to the manufacturer’s protocol. Cells were transfected again after 24 h to ensure the knockdown. On day 3 after transfection, BMDMs were challenged with a culture medium with or without cytochalasin D (10 μg/μL) (NSC 209835, MCE). An hour later, the same number of ACs consistent with the number of BMDMs were resuspended with the culture medium and added (or not) to the cells for 2–3 h. For the generation of ACs, H9C2 cells were cultured in DMEM with 10% FBS and 1% penicillin and streptomycin, and apoptosis was then induced under ultraviolet radiation for 2 hours.

Subsequently, mitochondria were extracted from BMDMs (about 1 × 10^7^) after being washed with ice-cold PBS twice, using a Mitochondria Isolation Kit for Cell Tissue (Yeasen Biotech Co., Ltd, China). Extraction of NAD^+^/NADH was performed with the buffers supplied within the commercial assay kits following the manufacturer’s instructions (Abcam ab65348 for NADH). Colorimetric measurements were made at 450 nm absorbance and 25 °C using the SpectraMax i3X (MD Co., Ltd, USA). NAD^+^ and NADH concentrations were determined on a standard curve according to the manufacturer’s instructions. The sequence of SLC25A53 siRNA is GGUGUCUGGUAGUGUCAAUTT (sense)/AUUGACACUACCAGACACCTT (anti-sense). The sequence of the SLC25A51 siRNA is CUCCAUUCGAAAGGGUUCATT (sense) / UGAACCCUUUCGAAUGGAGTT (anti-sense).

### Mitochondrial NAD^+^ uptake assay

BMDMs were extracted from WT mice and cultured for 5 days, then SLC25A53 was knockdown following the above steps. For the first transfection, 50 μg/mL uridine (HY-B1449, MCE) was added to the medium to improve cellular viability. 18-24 hours before harvest, the medium was changed, and the cells were treated with 100 nM FK866[(E)-Daporinad] (HY-50876, MCE). Cells were collected by trypsinization. Mitochondria were isolated as mentioned above and were resuspended (50–200 μg) in MiR05 (60101-01, NextGen-O2k) containing 5 mM malate (T2S0850, TargetMol) and 10 mM pyruvate (T4804, TargetMol) along with 1 mM NAD^+^ (HY-B0445, MCE). The reaction was agitated at 900 rpm and the tube was briefly opened every 10 min to allow for re-oxygenation. Mitochondria were granulated by centrifugation (14,000 × *g*, 2 min), and mitochondria NAD^+^ content was measured according to the manufacturer’s instructions (ab65348, Abcam).

### Molecular docking

Three-dimensional structures of SLC25A53 were designed with SWISS-MODEL (https://swissmodel.expasy.org/), and NAD^+^ was generated with RCSB Protein Data Bank. We used AutoDock Vina software (Trott & Olson, 2010) to perform the molecular docking between SLC25A53 and NAD^+^. Among the docking models generated, the model with the highest affinity was created with PyMol Molecular Graphics System (Schrodinger LLC, UK) and was shown in Fig. 5A.

### RNA-seq

CCR2^+^ macrophages within post-MI tissue were specially sorted out using FACS, and RNA of macrophages was extracted using the TRIzol reagent (15596018, Invitrogen) according to the manufacturer’s instructions. RNA integrity was assessed using the Agilent 2100 Bioanalyzer (Agilent Technologies, Santa Clara). Then the libraries were constructed using TruSeq Stranded mRNA LT Sample Prep Kit (Illumina, USA) according to the manufacturer’s instructions. The transcriptome sequencing and analysis were conducted by OE Biotech Co. Ltd. (Shanghai, China). FASTQ sequence data were mapped to mice reference genome NCBI Murine6.0 with TopHat v2.1.1. The reads per gene were counted using “HTseq” (https://htseq.readthedocs.io, version 0.6.0). Sample quality metrics and raw read counts were imported into R for further processing. The DESeq2 R package was used to estimate library size factors, normalize counts, and perform differential expression analyses. Benjamini–Hochberg multiple testing correction was used to compute FDR, and genes were considered significantly differentially expressed at <5% FDR. Principal component analysis (PCA) was performed in R using the top 1,000 most variable genes, with normalized DESeq2 variance-stabilized transformation expression as input. FASTQ files and expression matrices from RNA-seq data are available from the NCBI Gene Expression Omnibus (GEO) database under the accession number GSE218002.

### LC–MS analysis

BMDMs were generated and cultured for 5 days. Cells were then challenged by cell suspensions of ACs consistent with the amount of BMDMs for 2 h. After being washed with PBS twice, mitochondria were extracted using a Mitochondria Isolation Kit for Cell Tissue (Yeasen Biotech Co., Ltd, Shanghai, China). The following extraction of metabolites was instructed by Shanghai Applied Protein Technology Co., Ltd (China). Briefly, cold methanol/acetonitrile (1:1) extraction solvent was added to remove the protein and extract the metabolites. For absolute quantification of the metabolites, stock solutions of stable-isotope internal standards were added to the extraction solvent simultaneously. The mixture was collected and centrifuged at 14,000 × *g* for 20 min at 4 °C to collect the supernatant. The supernatant was dried in a vacuum centrifuge. For LC–MS analysis, the samples were re-dissolved in acetonitrile/water (1:1) solvent and centrifuged at 14,000 × *g* at 4 °C for 20 min, and then the supernatant was injected. Analyses were performed using a UHPLC (1290 Infinity LC, Agilent Technologies) coupled to a QTRAP MS (6500+, Sciex) according to the instructions. Polled quality control (QC) samples were set in the sample queue to evaluate the stability and repeatability of the system. MultiQuant or Analyst was used for quantitative data processing. The QCs were processed together with the biological samples. Metabolites in QCs with a coefficient of variation of less than 30% were denoted as reproducible measurements.

After sum-normalization, the processed data were uploaded before importing into SIMCA-P (version 14.1, Sweden), where it was subjected to multivariate data analysis, including Pareto-scaled principal component analysis and orthogonal partial least-squares discriminant analysis (OPLS-DA). The sevenfold cross-validation and response permutation testing was used to evaluate the robustness of the model. The projection value of each variable in the OPLS-DA model was calculated to indicate its contribution to the classification.

### Statistical analysis

Data were shown as mean ± SEM. A two-sided, unpaired Student’s *t*-test was utilized to analyze the difference between two groups in the case of normally distributed variables. Mann–Whitney *U* test was used in non-normally distributed variables. Differences across three or more groups were tested using one-way ANOVA, followed by a post hoc analysis with the Bonferroni test. *P* value < 0.05 was defined as statistical significance.

### Reporting summary

Further information on research design is available in the Nature Research Reporting Summary linked to this article.

### Supplementary information

supplementary figure

Supplemental Material - Original Blots

Reporting Summary

## Data Availability

The data analyzed during this study are included in this published article and the supplemental data files. Additional supporting data are available from the corresponding authors upon reasonable request.
