# Supplementary material for: TREM2 macrophage promotes cardiac repair in myocardial infarction by reprogramming metabolism via SLC25A53
Source: Cell Death Differ. 2024 Jan 5;31(2):239–53. doi: 10.1038/s41418-023-01252-8 (PMC10850484; doi:10.1038/s41418-023-01252-8)
Supplement: Supplementary file 1 — supplementary figure [file 41418_2023_1252_MOESM1_ESM.docx]

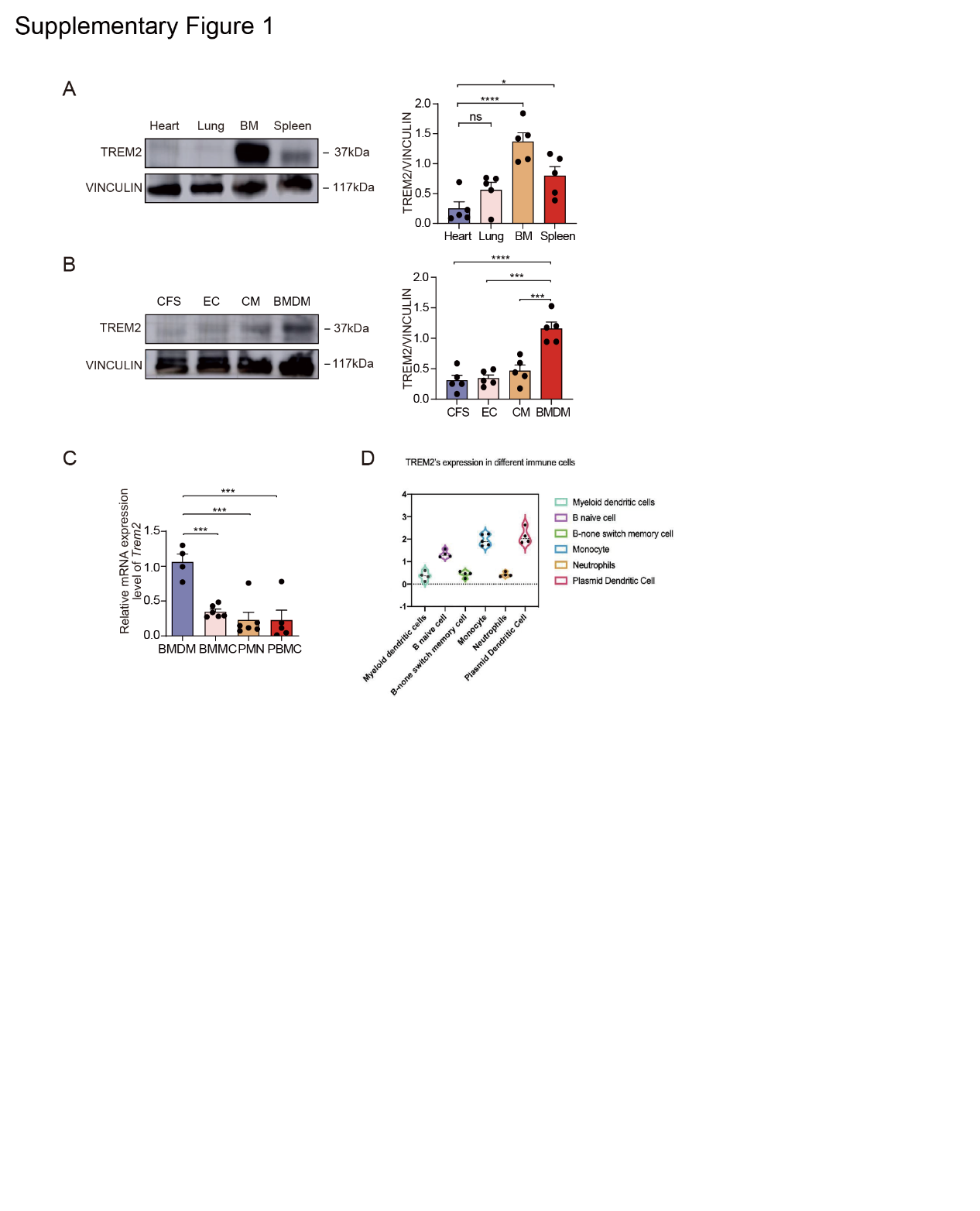


**A,** Trem2 expression was analyzed by western blot in heart, lung, spleen and bone marrow in w WT C57BL/6 mice (n=5). **B,** Western blot analyses of Trem2 expression in bone marrow derived macrophage, H9C2 cardiomyocytes, CFS fibroblasts and VEC endothelial cells, respectively (n=5). **C,** Trem2 expression was analyzed in bone marrow derived macrophage, bone marrow mononuclear cell, polymorphonuclear and peripheral blood mononuclear cells by quantitative polymerase chain reaction (n=4-6). **D,** Trem2 expression was analyzed by bioinformatics analysis in different immune cells(n=4).


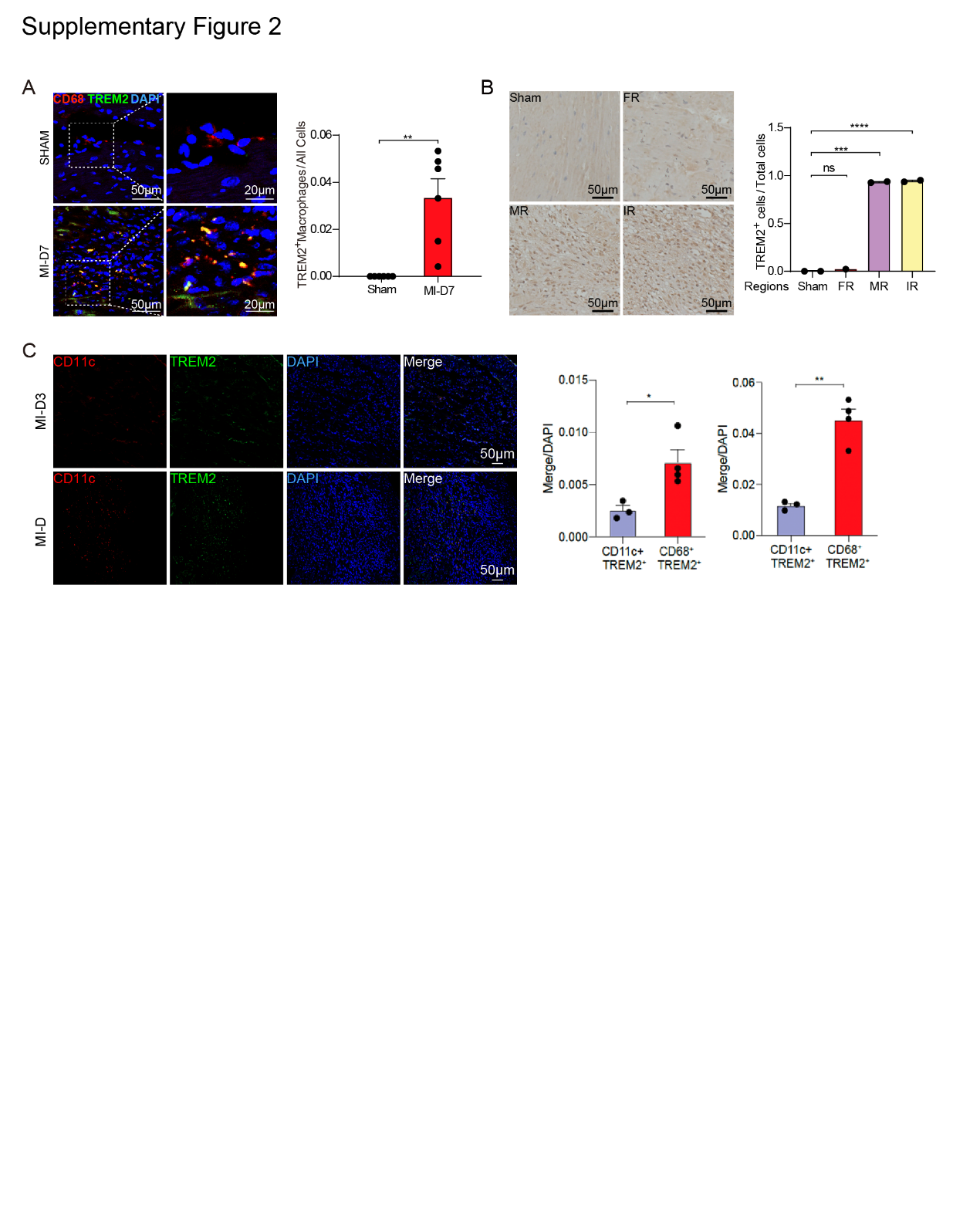


**A,** Representative immunofluorescence images and related quantification of CD68 and TREM2 in heart of control mice with sham operation and on Day 7 post-MI. **B,** Representative images of IHC test of TREM2 in different regions of groups described in **A. C,** Representative immunofluorescence images of CD11c and TREM2 in heart of control mice and related quantification Day 3(left) and Day 7 post-MI (right)**.** N=2-6 per group. Data were expressed as mean± SEM. Date in B was analyzed by one-way ANOVA followed by Bonferroni post hoc analysis. Other data were analyzed by Mann-Whitney U tests. ns indicates not significant. *P<0.05. **P<0.01. ***P<0.001.


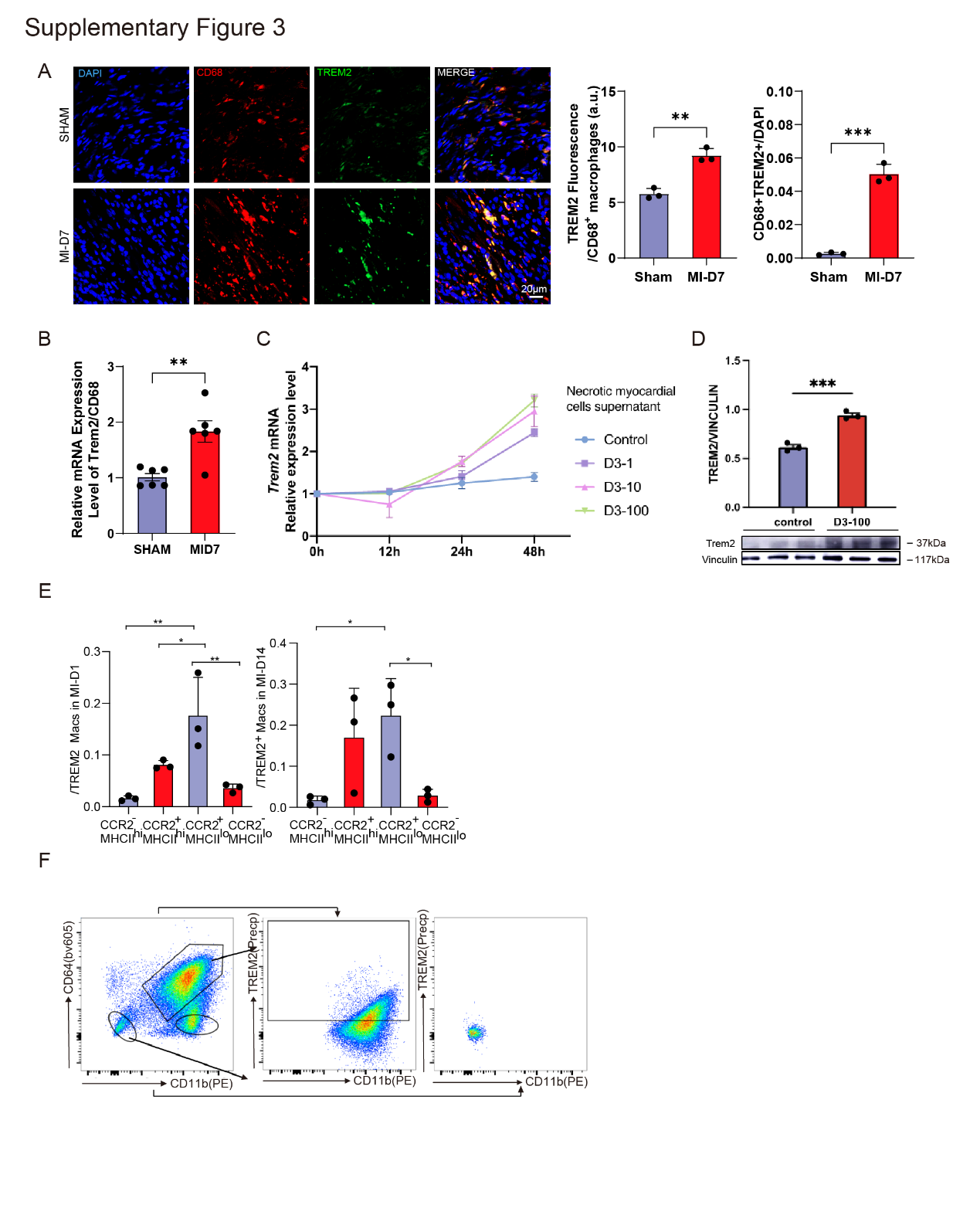


**A,** Immunofluorescence images of CD68 and TREM2 in heart of control mice and those on day 7 post-MI. TREM2 fluorescence was quantified per CD68^+^ macrophage (middle). CD68^+^TREM2^+^ cells were quantified per DAPI^+^ cell (right). **B,** Trem2 expression was analyzed in macrophages isolated from heart of healthy mice and those on day 7 post-MI by quantitative polymerase chain reaction (n=6). **C,** Trem2 expression was analyzed in RAW264.7 cells by quantitative polymerase chain reaction (n=3). **D,** Trem2 expression was analyzed by western blot in RAW264.7 cells (n=3). In **C&D,** “D3-1” refers to the necrotic cardiomyocyte cells supernatant 3 days after myocardial infarction, with a stimulation concentration of 1 ug/ml. “D3-10” refers to the necrotic cardiomyocyte cells supernatant 3 days after myocardial infarction, with a stimulation concentration of 10ug/ml. “D3-100” refers to the necrotic cardiomyocyte cells supernatant 3 days after myocardial infarction, with a stimulation concentration of 100 ug/ml. **E,** Flow cytometric analysis of different subsets in TREM2^+^ macrophages in heart of control mice on Day 1 and Day 14 post-MI (n=3)**. F,** Gating strategy of flow cytometry described in **Figure H-I and Supplementary Figure 2A.** Data were expressed as mean± SEM. Data were analyzed by one-way ANOVA followed by Bonferroni post hoc analysis. ns indicates not significant. *P<0.05. **P<0.01. ***P<0.001.


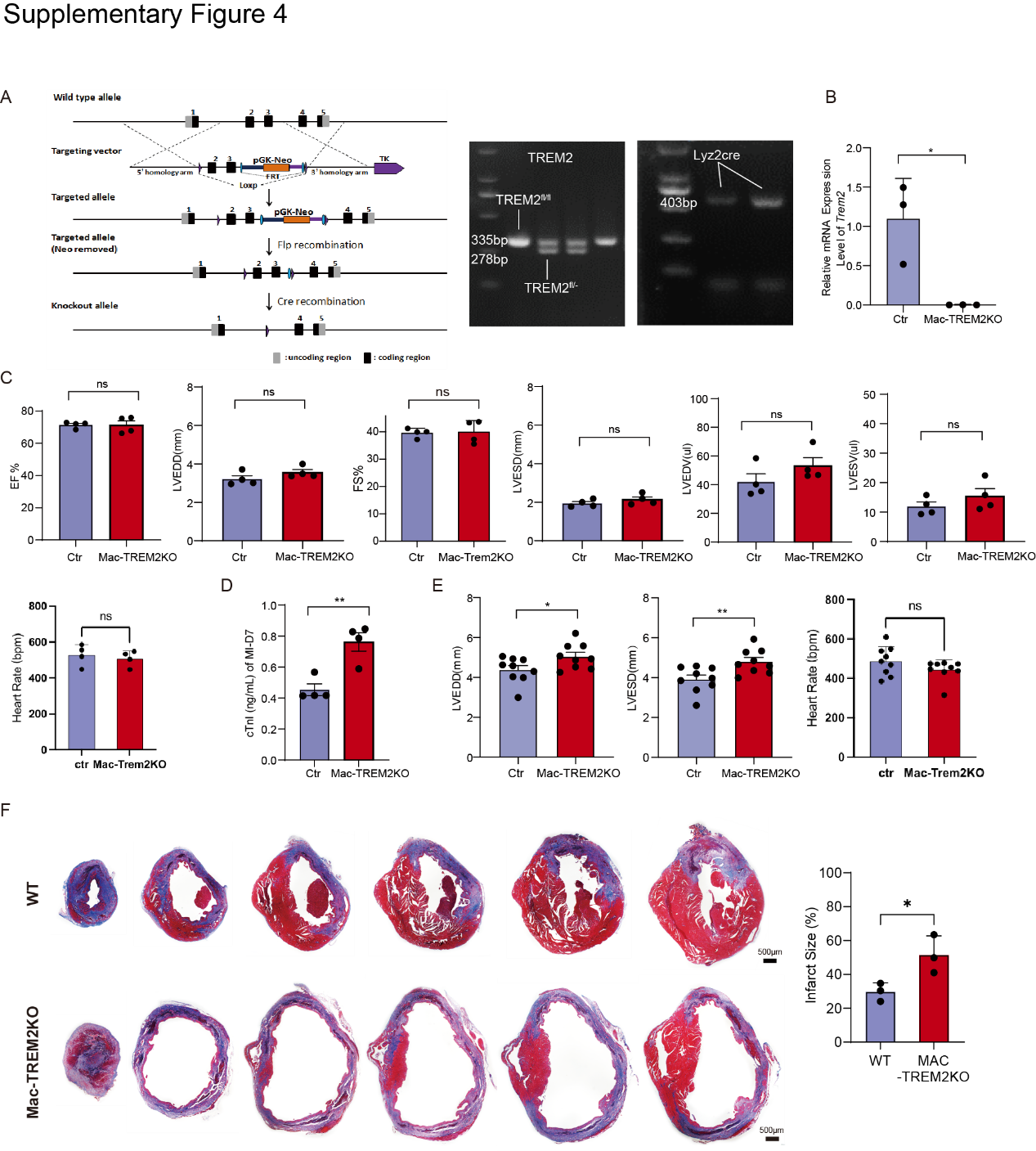


**A,** Schematic diagram of TREM2 gene-trapping strategy. Representative PCR genotyping of wild-type (+/+), homozygous (-/-), and heterozygous (+/-) mutant mice. TREM2 homozygous generated one fragment (335 bp), whereas heterozygous generated two fragments (335 bp and 278 bp) in PCR. LysM would take effect in the existence of the Cre enzyme. **B,** mRNA expression level of TREM2 in spleen of control and Mac-TREM2KO mouse (n=3). **C,** Echocardiographic measurements in control and Mac-TREM2KO mice prior to MI surgery (n=3). **D,** The concentration of serum cTnI (ng/mL) of control and Mac-TREM2KO mice on Day 7 post-MI (n=3). **E,** Echocardiographic analysis of LVEDD and LVESD quantification in control and Mac-TREM2KO mice on Day 7 post-MI (n=9). **F,** Representative images and related quantification of Masson’s trichrome staining on a series of sections over the entire heart in WT and Mac-TREM2KO mice (n=3). Data were expressed as mean± SEM. Data were analyzed by Mann-Whitney U tests. ns indicates not significant. *P<0.05. **P<0.01. ***P<0.001.


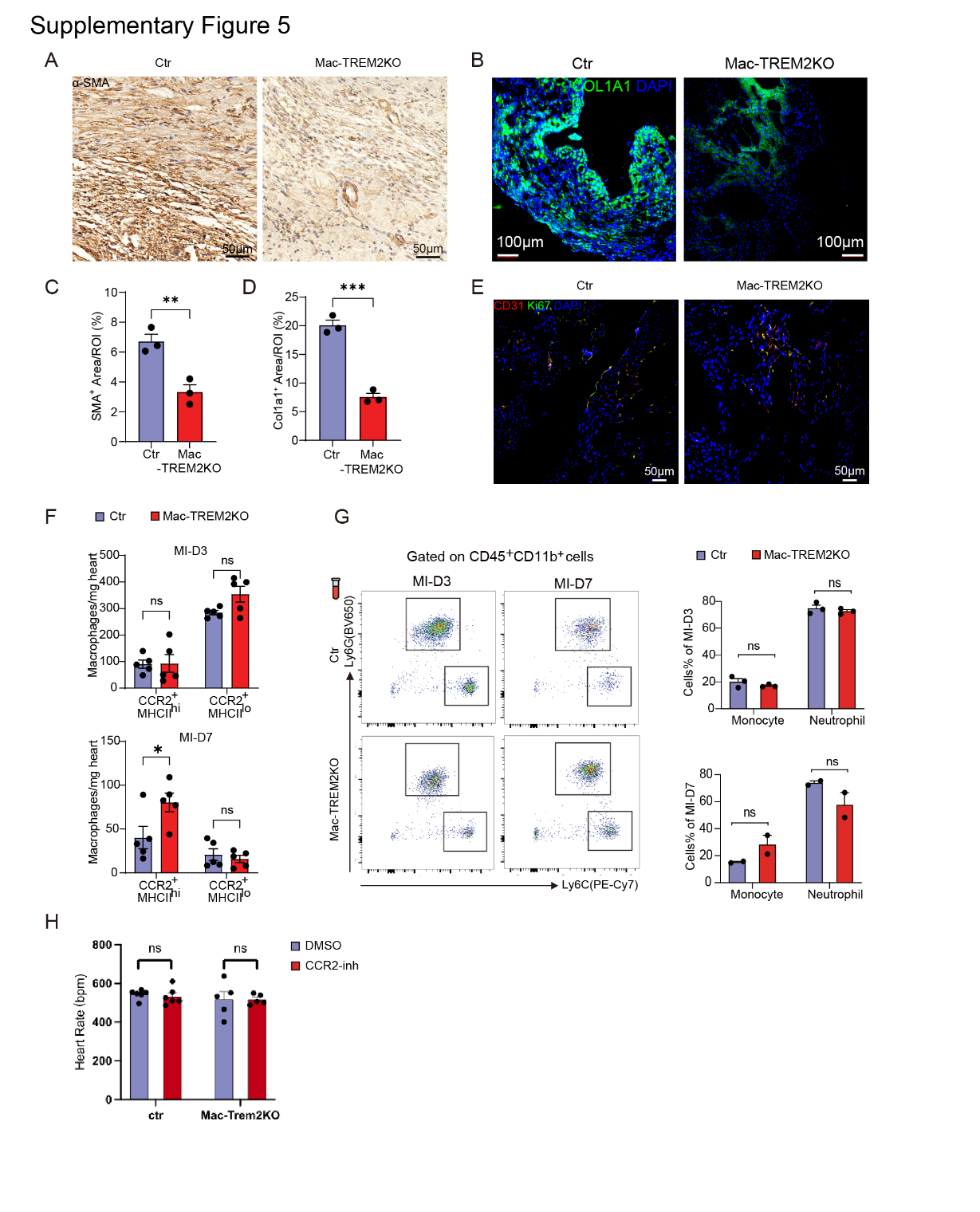


**A**, Representative images of IHC staining of α-SMA in the infarcted myocardium from control and Mac-TREM2KO mice. **B**, Representative immunofluorescence images of Col1a1 in the infarcted myocardium from control and Mac-TREM2KO mice. **C&D**, Related quantification of **A&B** respectively (n=3). **E**, Representative immunofluorescence staining of Ki67^+^ CD31^+^ cells in the infarcted myocardium from control and Mac-TREM2KO mice. **F,** Related quantification of number of macrophages per mg heart within infarcted myocardium of control and Mac-TREM2KO mice on Day 3 and Day 7 post-MI (n=5). **G**, Gating strategy of monocyte and neutrophil on CD45^+^ CD11b^+^ cells in peripheral blood in control or Mac-TREM2KO mice(left) and related flow cytometric analysis for the percentage of monocytes and neutrophils (right) (n=2-3). **H,** Heart rate analysis of echocardiographic measurements in **Figure 2M&N**. Data were expressed as mean± SEM. Data in C was analyzed by Mann-Whitney U tests. Other data were analyzed by 2-way ANOVA followed by Bonferroni post hoc analysis. ns indicates not significant. *P<0.05. **P<0.01. ***P<0.001


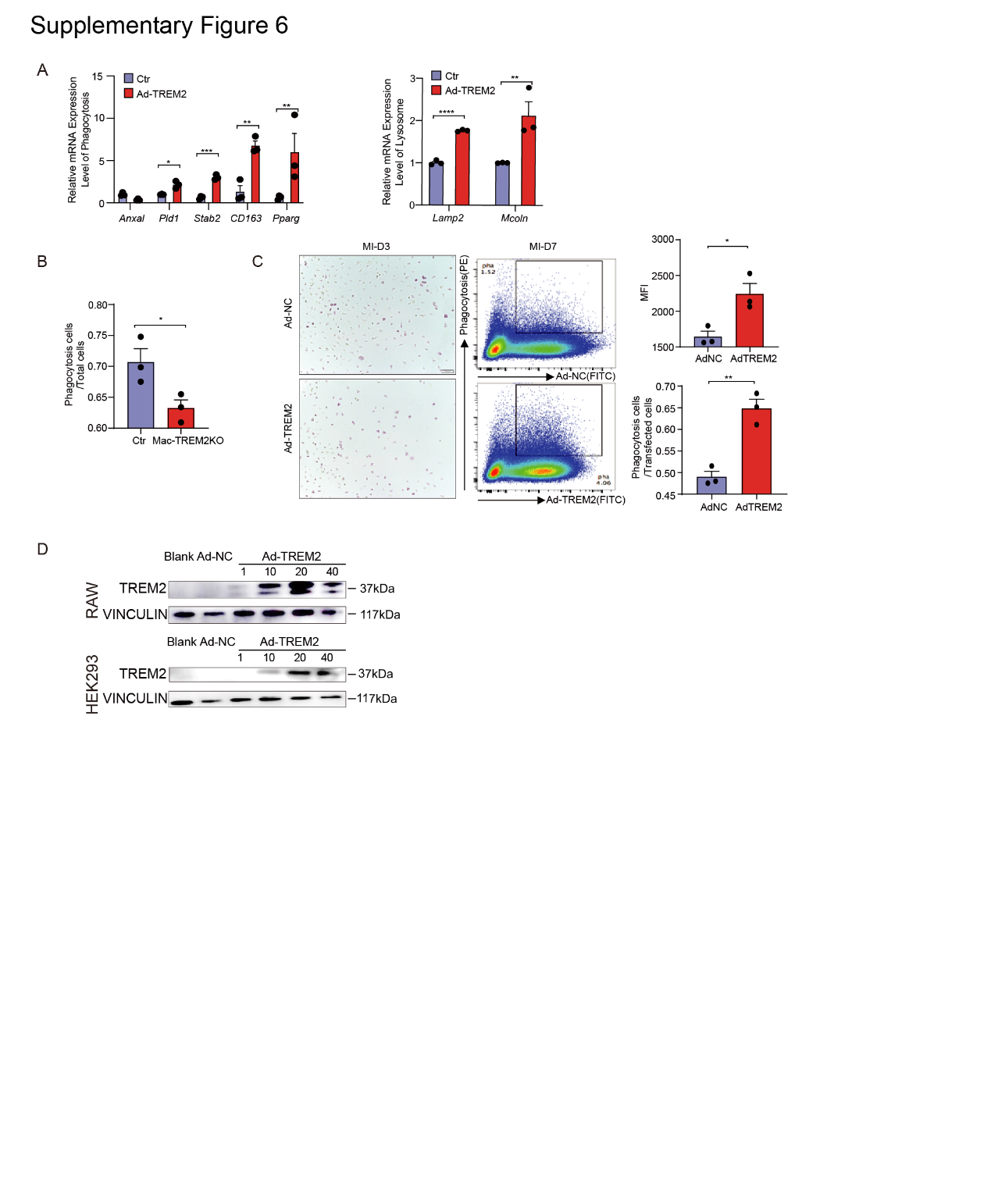


**A**, Relative mRNA expression levels of phagocytosis-related genes and lysosome related genes in control BMDMs treated with Ad-NC and Ad-TREM2. **B,** Flow cytometric analysis of phagocytosis cells in control and TREM2KO BMDMs incubated with Red Zymosan Bioparticles. **C,** Flow cytometry and Mean Fluorescence Intensity (MFI) of phagocytosis cells in BMDMs treated with Ad-NC and Ad-TREM2 and then incubated with Red Zymosan Bioparticles. **D,** Western blot analysis of validation of TREM2 overexpression by adenovirus in RAW and HEK293 cells. N=3 per group. Data were expressed as mean± SEM. Data in A was analyzed by 2-way ANOVA followed by Bonferroni post hoc analysis. Other data were analyzed by Mann-Whitney U tests. ns indicates not significant. *P<0.05. **P<0.01. ***P<0.001


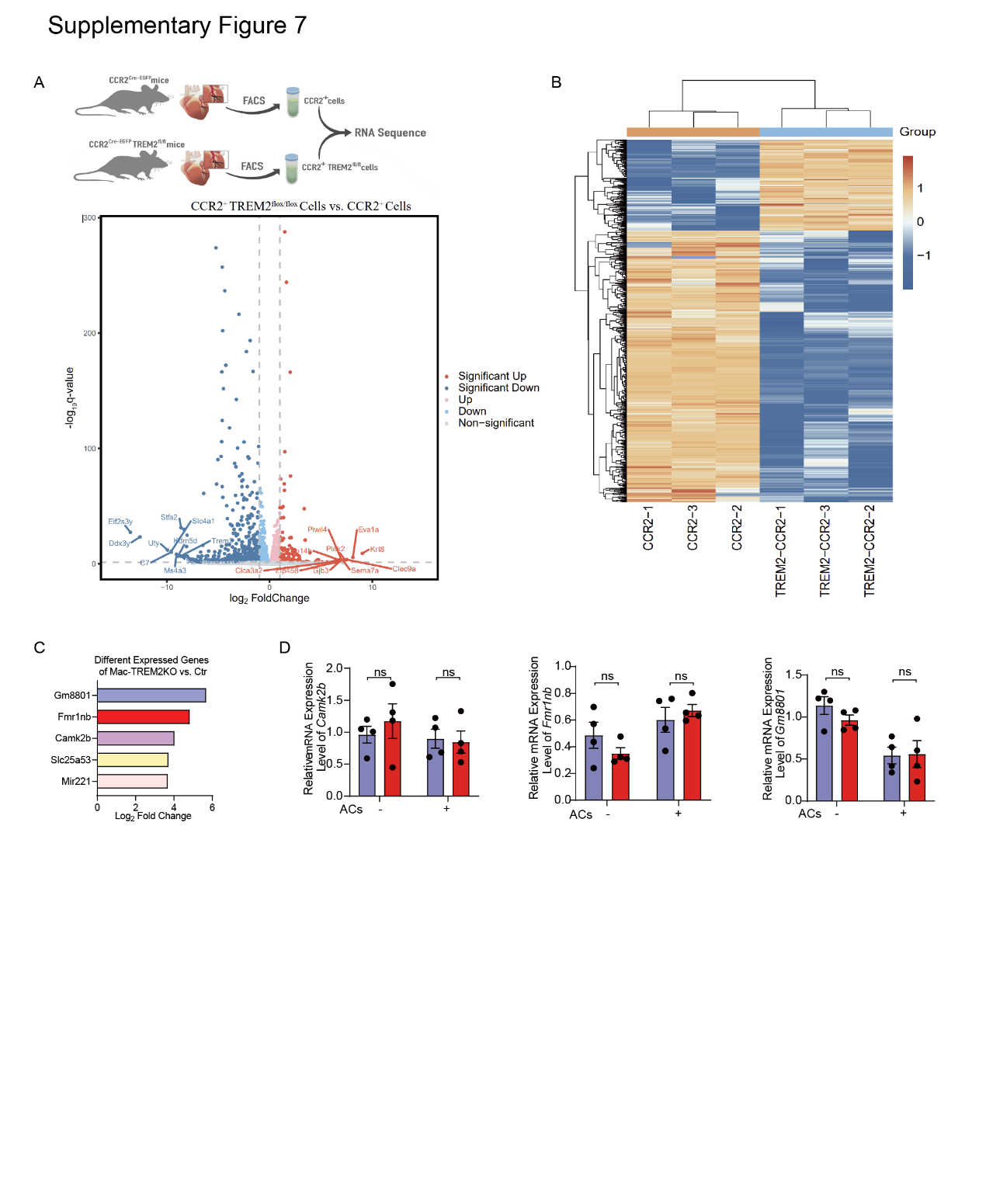


**A**, Diagram of cell sorting for RNA-seq and volcano plot of RNA-seq results. **B**, Heatmap results of RNA-seq results. **C**, Differently expressed genes of Mac-TREM2KO vs control ranked by Log_2_ Fold Change. **D,** Relative mRNA expression of related genes in **C** verified by qPCR (N=4). Data were expressed as mean± SEM. Data were analyzed by 2-way ANOVA followed by Bonferroni post hoc analysis. ns indicates not significant.


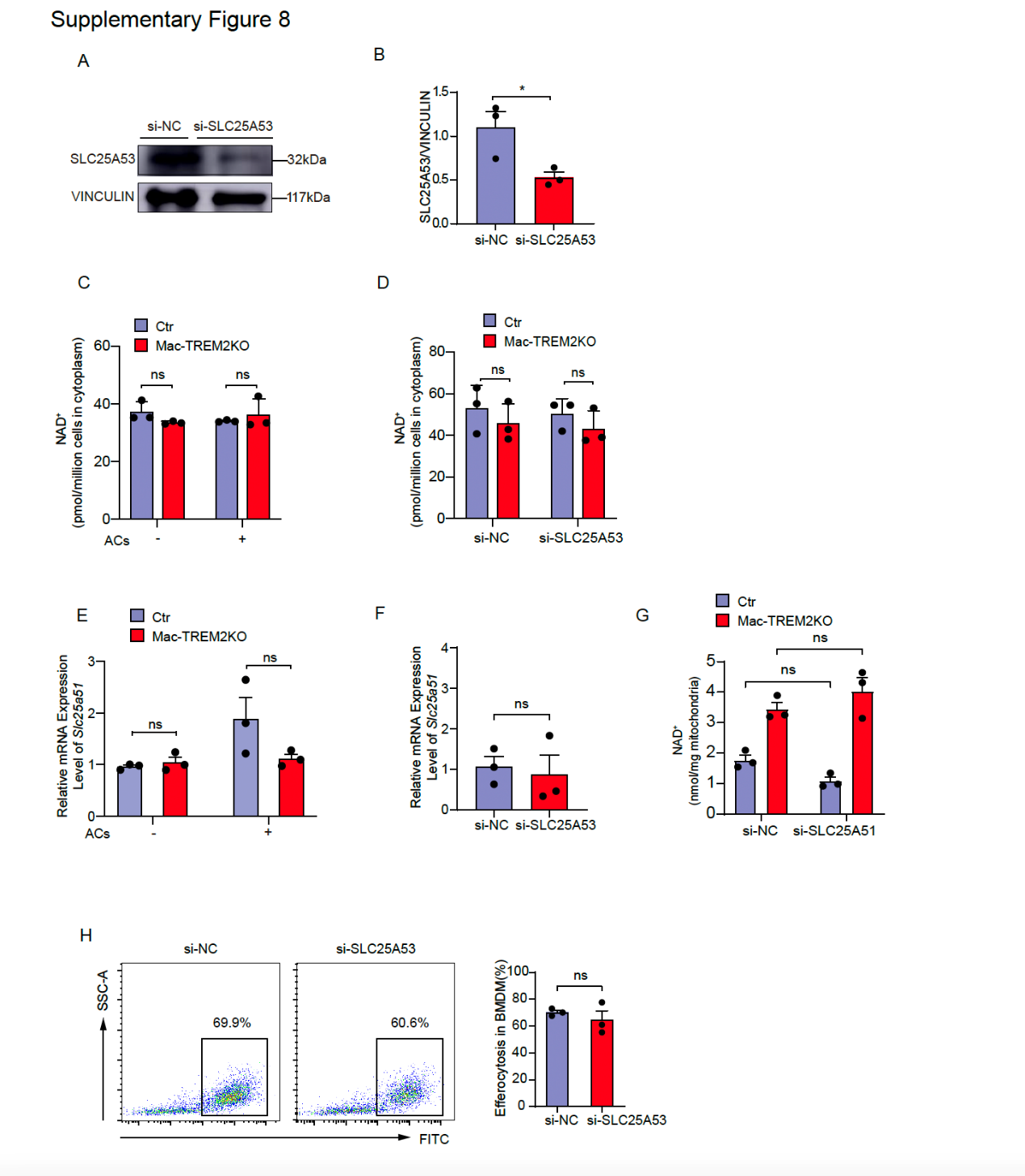


**A&B**, Western blot analysis showed the successful transfection of siRNA to inhibit the expression of SLC25A53. **C**, Quantification of NAD^+^ concentration in the cytoplasm of BMDMs extracted from control and Mac-TREM2KO mice. **D**, Quantification of NAD^+^ concentration in the cytoplasm of si-NC and si-SLC25A53 BMDMs extracted from control and Mac-TREM2KO mice. **E**, qPCR for the relative mRNA expression of SLC25A51 in control and TREM2KO BMDMs with or without the co-culture of ACs. **F**, Relative mRNA expression of SLC25A51 in BMDMs treated with si-NC or si-SLC25A53. **G**, Quantification of NAD^+^ content in isolated mitochondria in control and TREM2KO BMDMs transfected with si-NC or si-SLC25A51. **H,** Flow cytometric analysis of efferocytosis abilities in BMDMs transfected with si-NC or si-SLC25A51 and incubated with GFP^+^ ACs. N=3 per group. Data were expressed as mean± SEM. Data in B&F&H were analyzed by Mann-Whitney U tests. Other data were analyzed by 2-way ANOVA followed by Bonferroni post hoc analysis. ns indicates not significant. *P<0.05. **P<0.01. ***P<0.001.


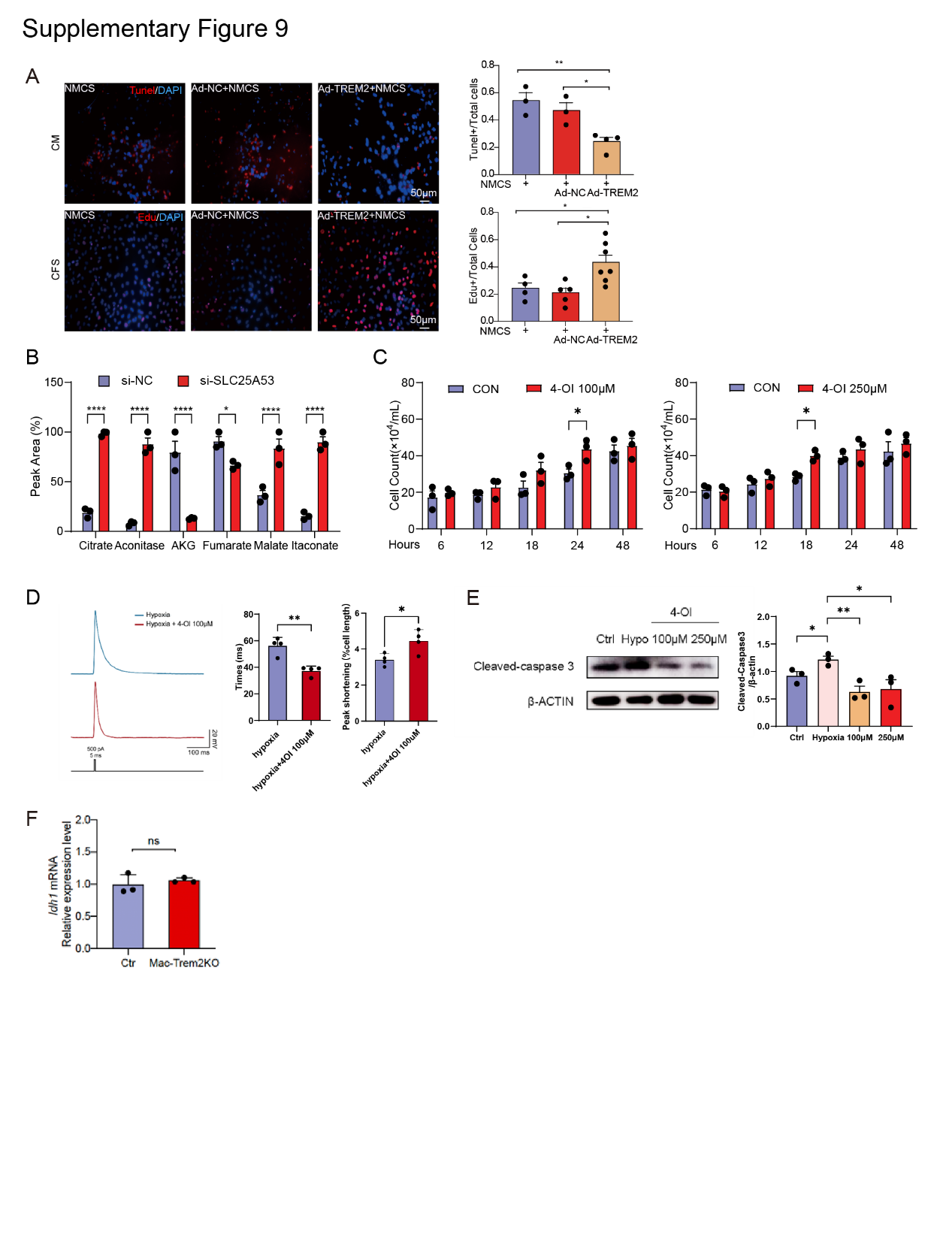


**A,** Representative immunofluorescence images of EDU+ CFS AND TUNEL+ CM after treatment with supernatants from BMDMs treated with Ad-NC or Ad-TREM2 that was preincubated with NMCS (n=3-7). **B,** Relative quantification of different metabolites content analyzed by LC-MS in mitochondria of BMDMs treated with si-NC or si-SLC25A53 (n=3). **C,** Cell counting of neonatal rat’s cardiomyocytes stimulated with or without 100μM or 250μM 4-OI. **D,** Examples of action potential (AP) of a neonatal rat’s cardiomyocyte stimulated with electric current at 5ms, 500Pa (left), and analysis of AP duration (middle) and peak shortening per cell length (right) (n=4). **E,** Cleaved-caspase 3 expression was analyzed by western blot in hiPSCs-derived cardiomyocytes under low oxygen and low serum environment with or without 4-OI treatment (n=3). **F,** Relative mRNA expression of *Idh1* was analyzed by qPCR in control and TREM2KO BMDMs (n=3). Data were expressed as mean± SEM. Data in A was analyzed by one-way ANOVA followed by Bonferroni post hoc analysis. Data in B was analyzed by 2-way ANOVA followed by Bonferroni post hoc analysis. Data in C was analyzed by Mann-Whitney U tests. ns indicates not significant. *P<0.05. **P<0.01. ***P<0.001.


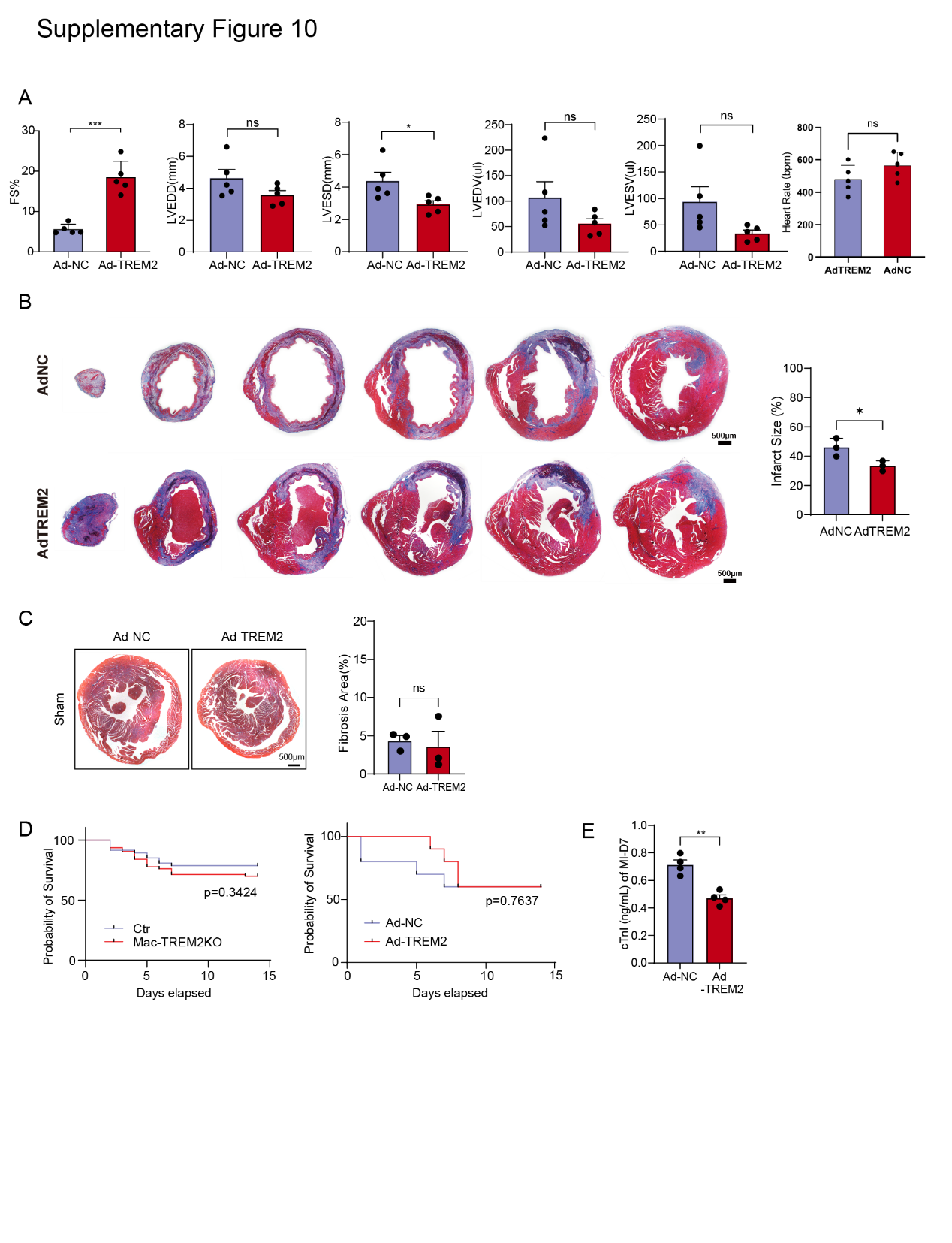


**A,** Echocardiographic measurements in groups described in **Figure 6A** (n=5). **B,** Representative images and related quantification of Masson’s trichrome staining in mice administered with Ad-NC or Ad-TREM2 immediately after surgery on day 7 post-MI (n=3). **C,** Representative images and related quantification of Masson’s trichrome staining in mice administered with Ad-NC or Ad-TREM2 under sham operation (n=3). **D,** Survival curve of control and Mac-TREM2KO mice post-MI (left) and mice treated with Ad-NC and Ad-TREM2 post-MI (right). **E,** Concentration of serum cTnI (ng/mL) in groups described in **Figure 6A** (n=3). Data were expressed as mean± SEM. Data were analyzed by Mann-Whitney U tests. ns indicates not significant. *P<0.05. **P<0.01. ***P<0.001.


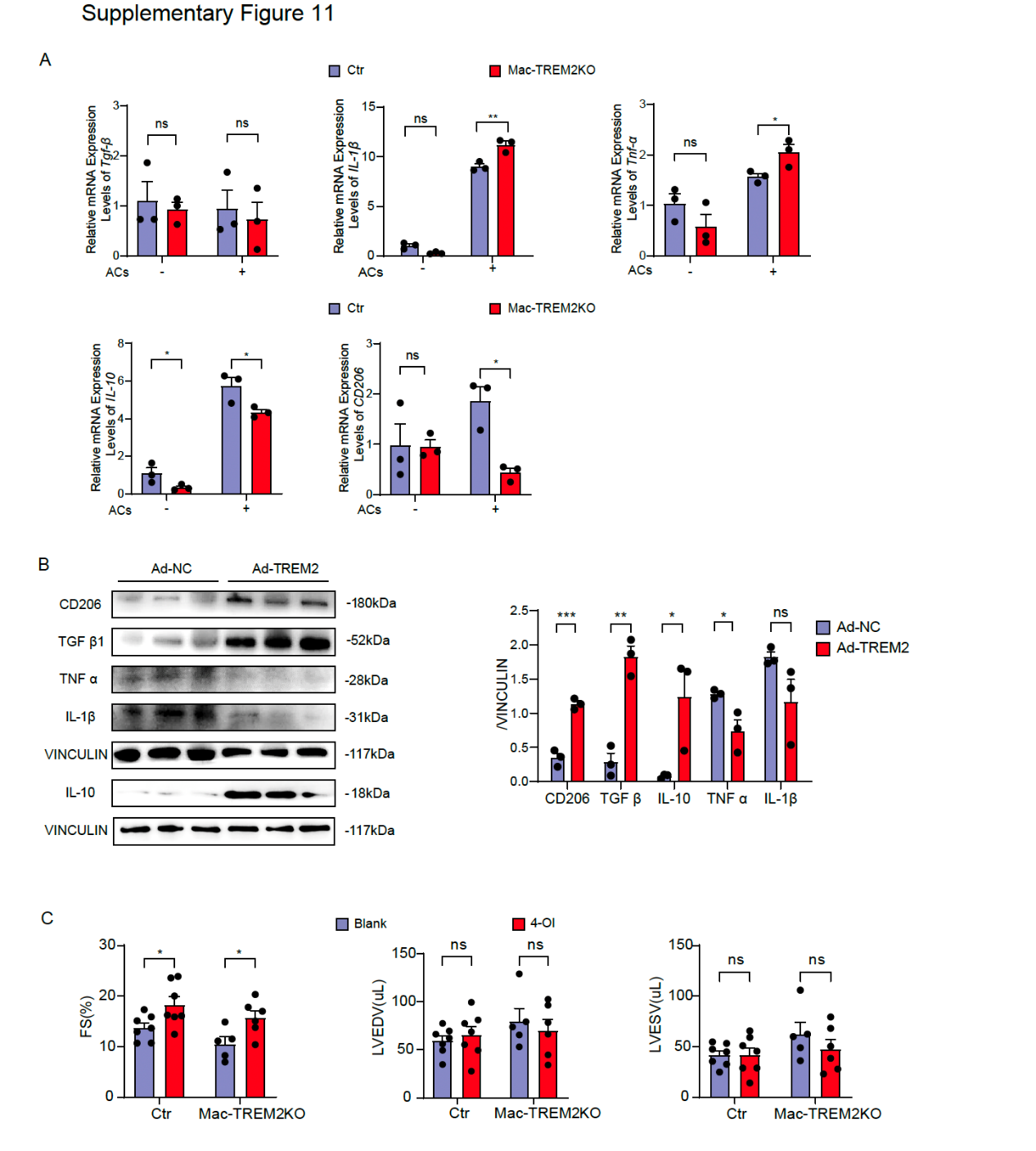


**A,** Relative mRNA expression of Tgf-β, IL-1β, Tnf-α, IL-10 and CD206 in control and TREM2KO BMDMs incubated with or without ACs (n=3). **B,** Western blot analysis of CD206, TGF-β1, TNF-α, IL-1β AND IL-10 in heart of mice treated with Ad-NC or Ad-TREM2 post-MI (n=3). **C,** Echocardiographic measurements in groups described in **Figure 6I** (n=5-7)**.** Data were expressed as mean± SEM. Data were analyzed by 2-way ANOVA followed by Bonferroni post hoc analysis. ns indicates not significant. *P<0.05. **P<0.01. ***P<0.001

Supplementary Table 1

|  | Control (n=10) | MI (n=29) | P value |
| --- | --- | --- | --- |
| male, % | 7 (70) | 24 (82.8) | 0.389 |
| Age, years | 62.70±6.83 | 63.69±10.18 | 0.994 |
| Diabetes % | 2 (20) | 12 (41.4) | 0.224 |
| Hypertension, % | 4 (40) | 18 (62.1) | 0.225 |
| EF, % | 62.60±2.17 | 48.17±7.64 | <0.0001 |
