# Supplementary material for: TREM2 macrophage promotes cardiac repair in myocardial infarction by reprogramming metabolism via SLC25A53
Source: Cell Death Differ. 2024 Jan 5;31(2):239–53. doi: 10.1038/s41418-023-01252-8 (PMC10850484; doi:10.1038/s41418-023-01252-8)

Figure1B

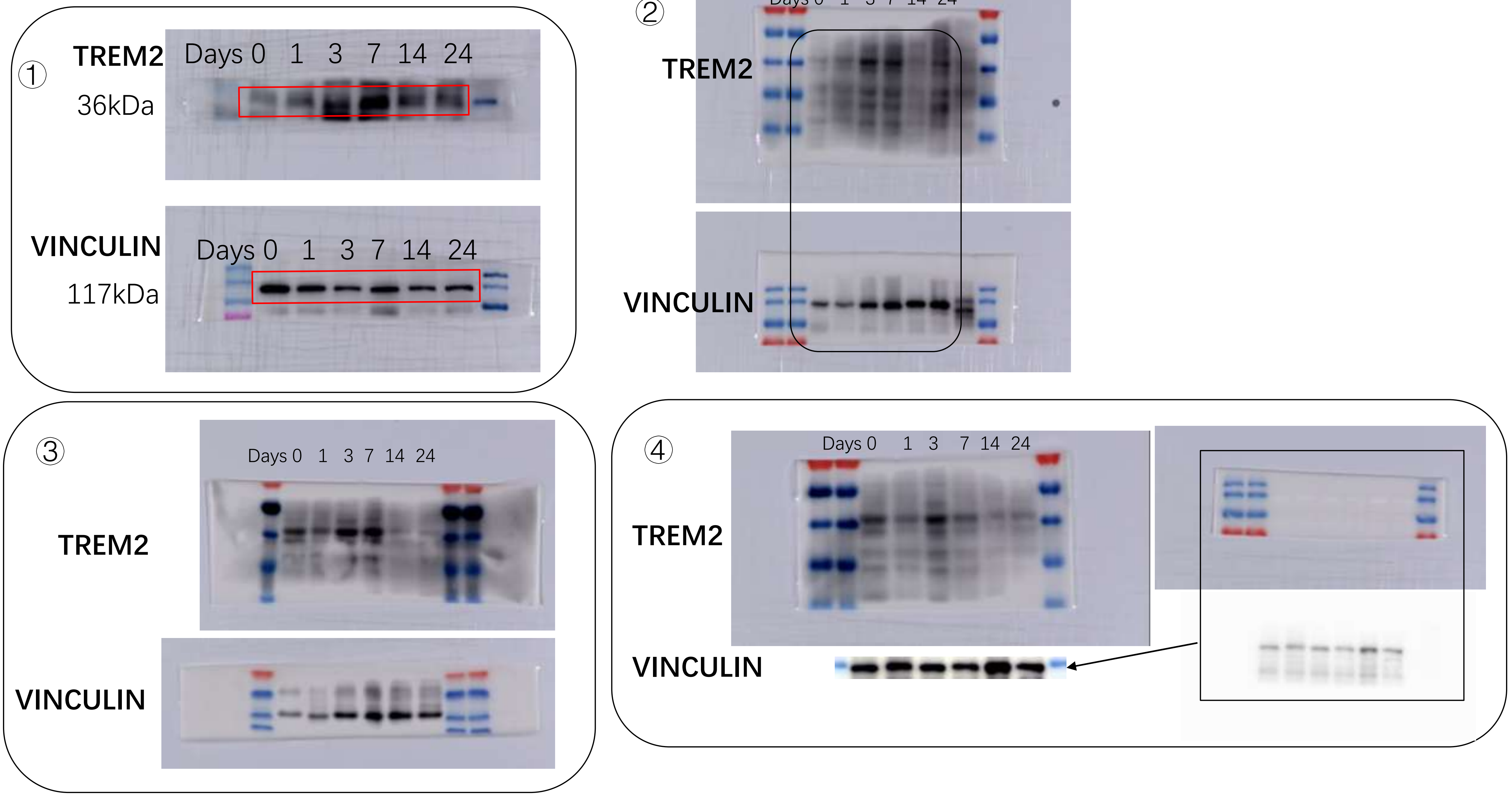

①
TREM2
36kDa
Days 0 1 3 7 14 24
VINCULIN
117kDa
Days 0 1 3 7 14 24
②
Days 0 1 3 7 14 24
TREM2
VINCULIN
③
Days 0 1 3 7 14 24
TREM2
VINCULIN
④
Days 0 1 3 7 14 24
TREM2
VINCULIN

Figure1B

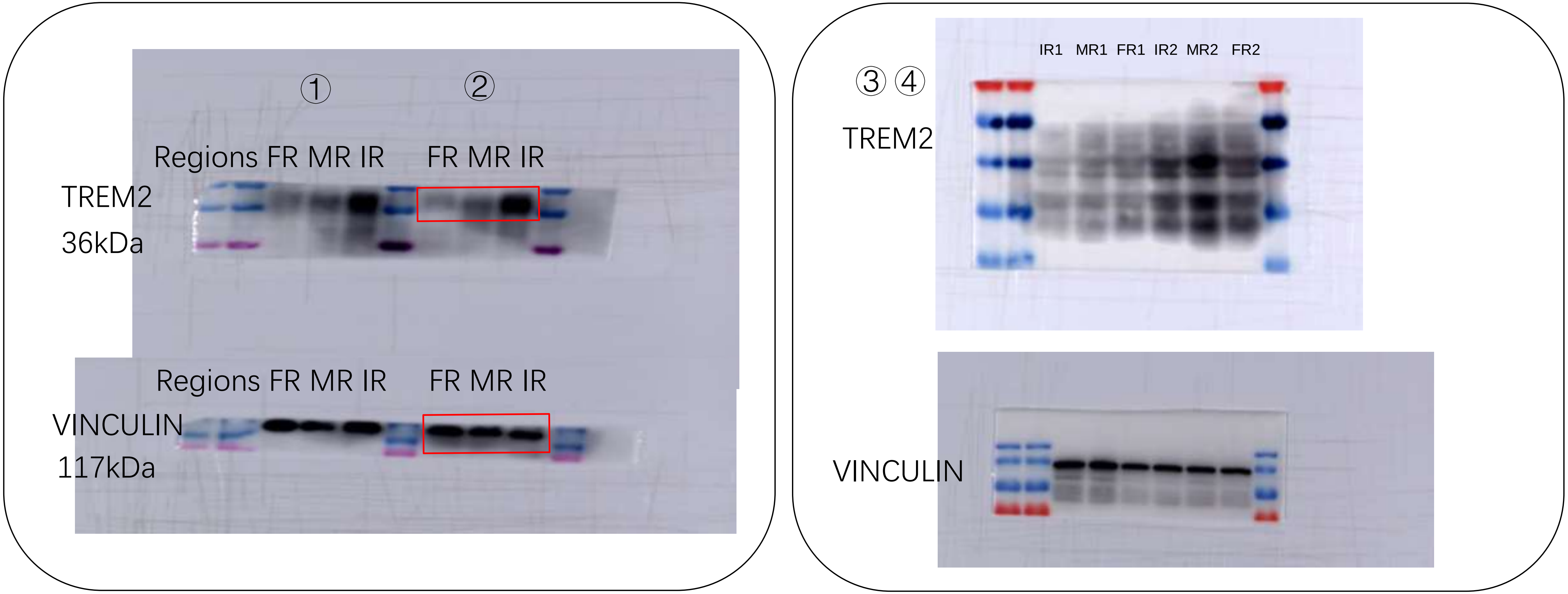
①
②
Regions FR MR IR
FR MR IR
TREM2
36kDa
Regions FR MR IR
FR MR IR
VINCULIN
117kDa
③ ④
IR1 MR1 FR1 IR2 MR2 FR2
TREM2
VINCULIN

Figure4D ①

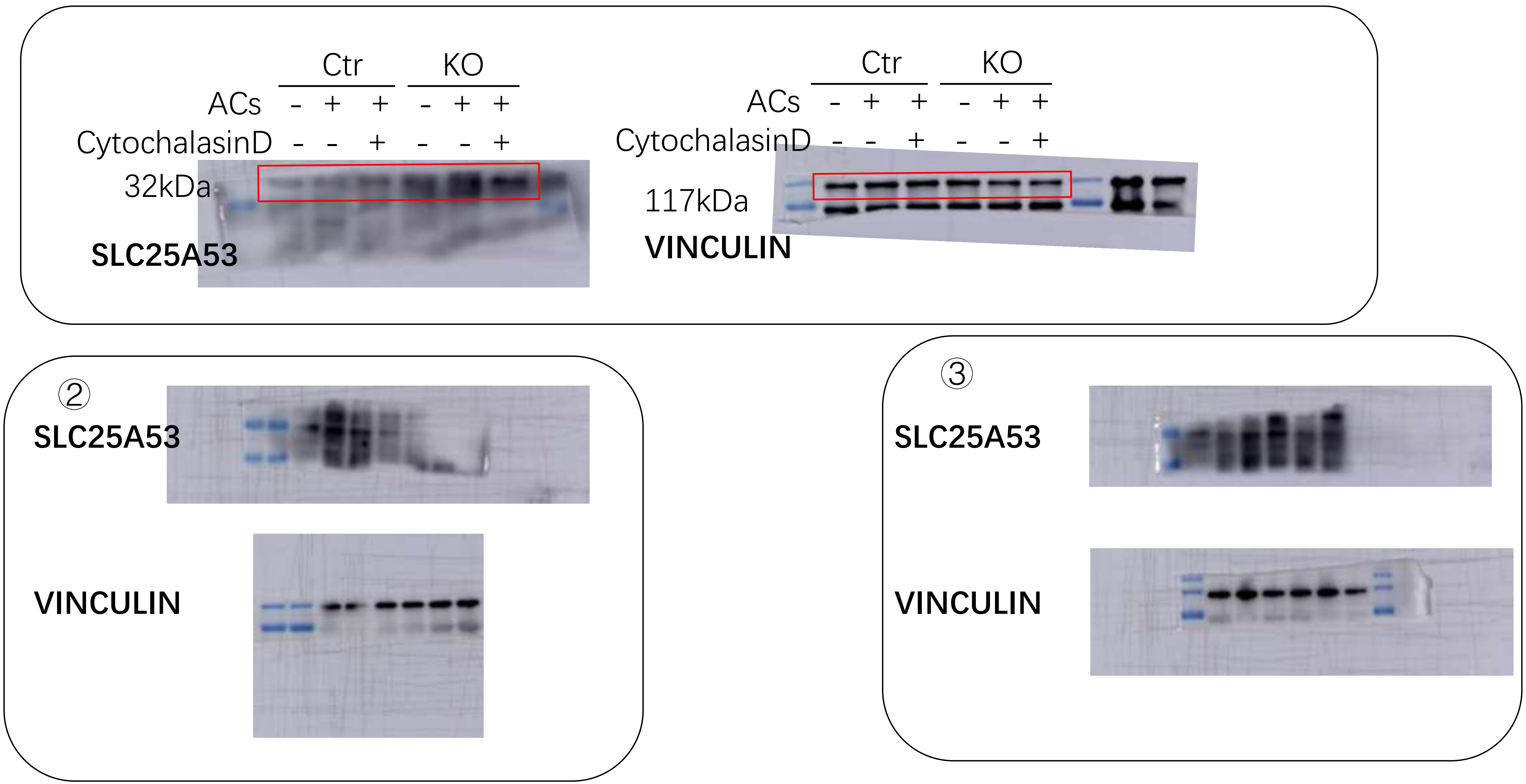

Ctr
KO
ACs - + + - + +
CytochalasinD - - + - - +
32kDa
SLC25A53
Ctr
KO
ACs - + + - + +
CytochalasinD - - + - - +
117kDa
VINCULIN
②
SLC25A53
VINCULIN
③
SLC25A53
VINCULIN

Figure4F ①

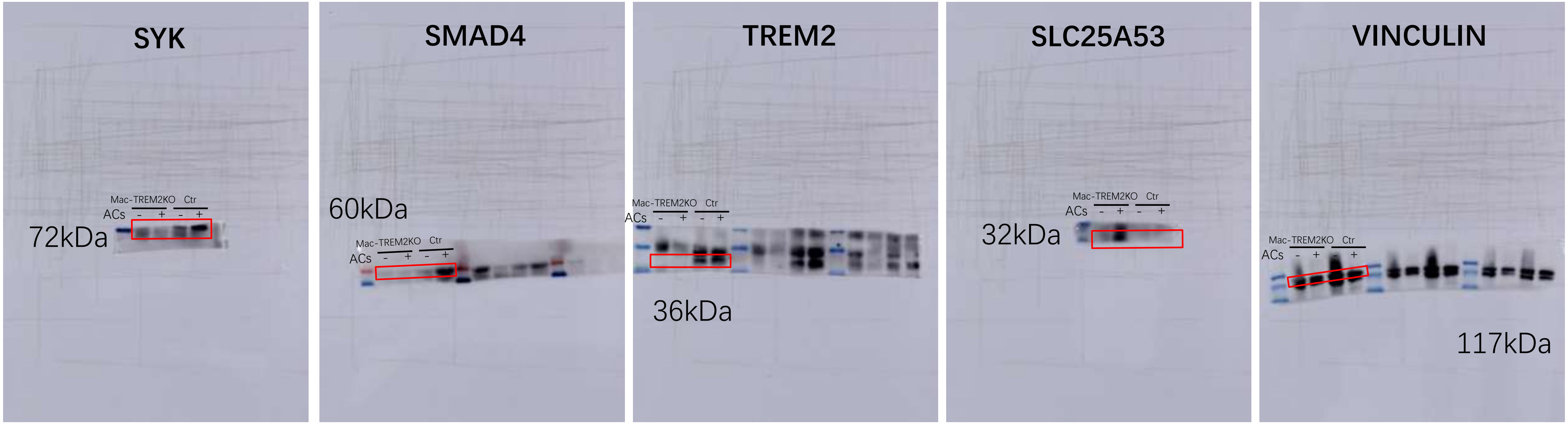

SYK
Mac-TREM2KO Ctr
ACs - + - +
72kDa
SMAD4
60kDa
Mac-TREM2KO Ctr
ACs - + - +
TREM2
Mac-TREM2KO Ctr
ACs - + - +
36kDa
SLC25A53
Mac-TREM2KO Ctr
ACs - + - +
32kDa
VINCULIN
Mac-TREM2KO Ctr
ACs - + - +
117kDa

Figure4F

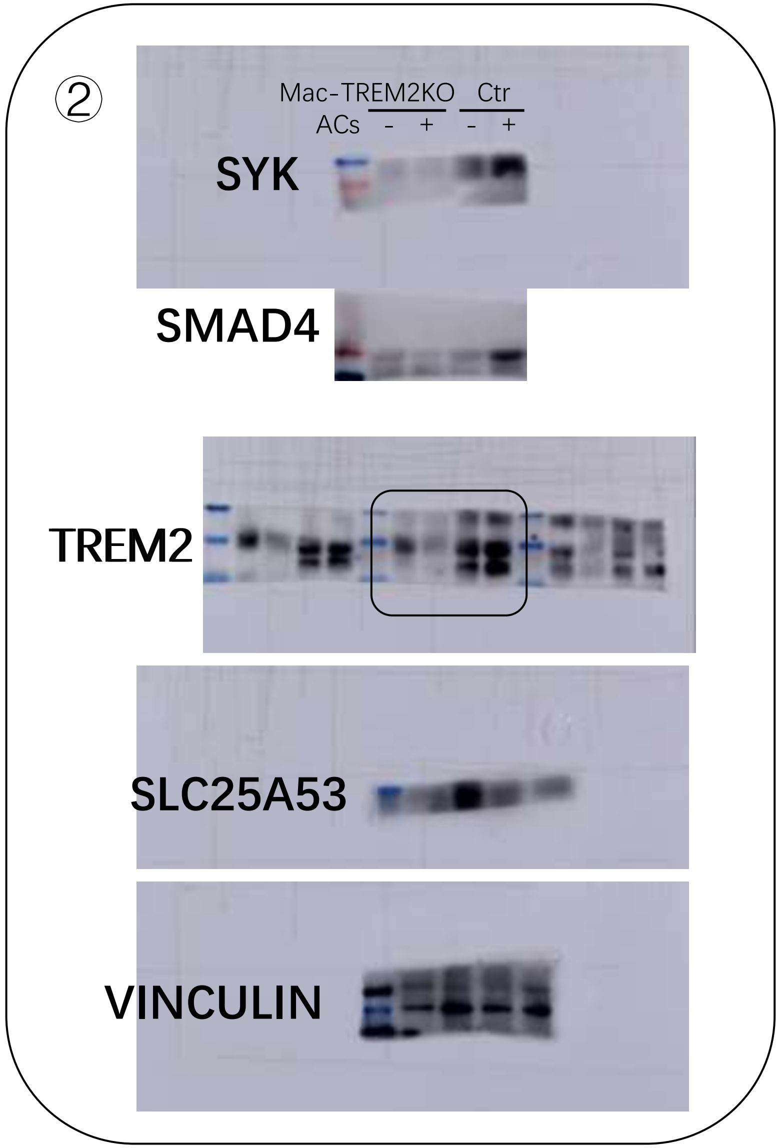
②
Mac-TREM2KO
Ctr
ACs
-
+
-
+
SYK
SMAD4
TREM2
SLC25A53
VINCULIN

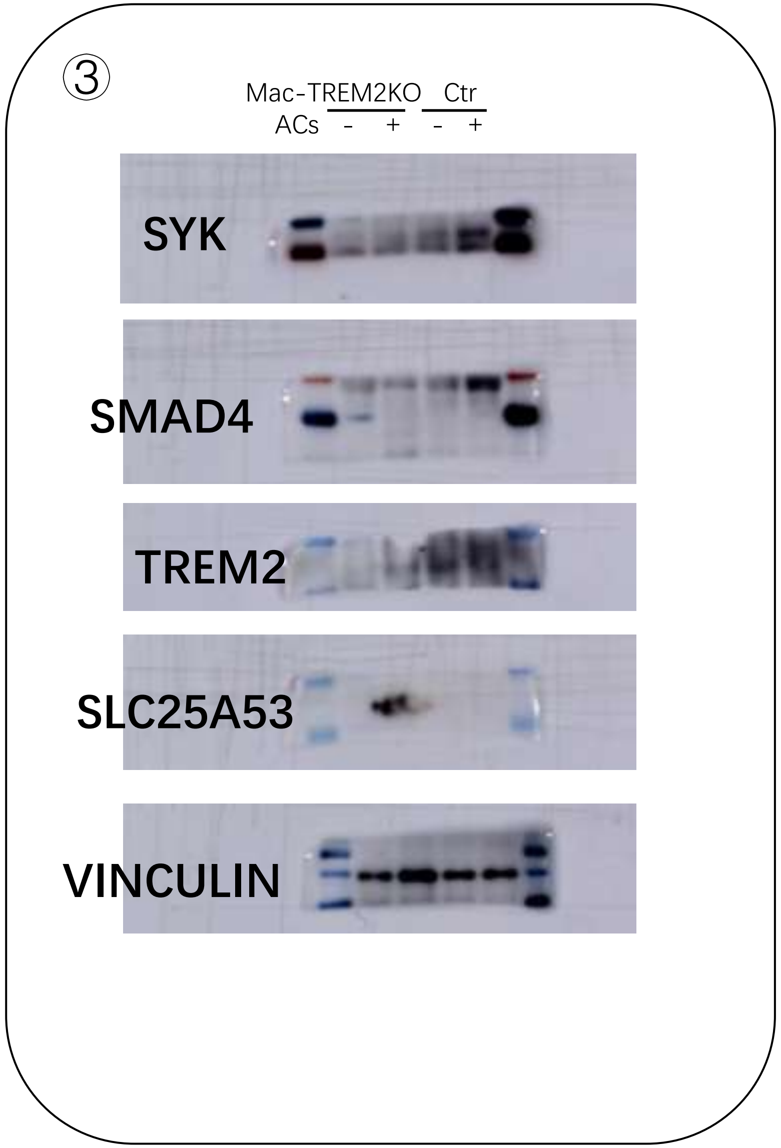
③
Mac-TREM2KO
Ctr
ACs
-
+
-
+
SYK
SMAD4
TREM2
SLC25A53
VINCULIN

Figure4G

①

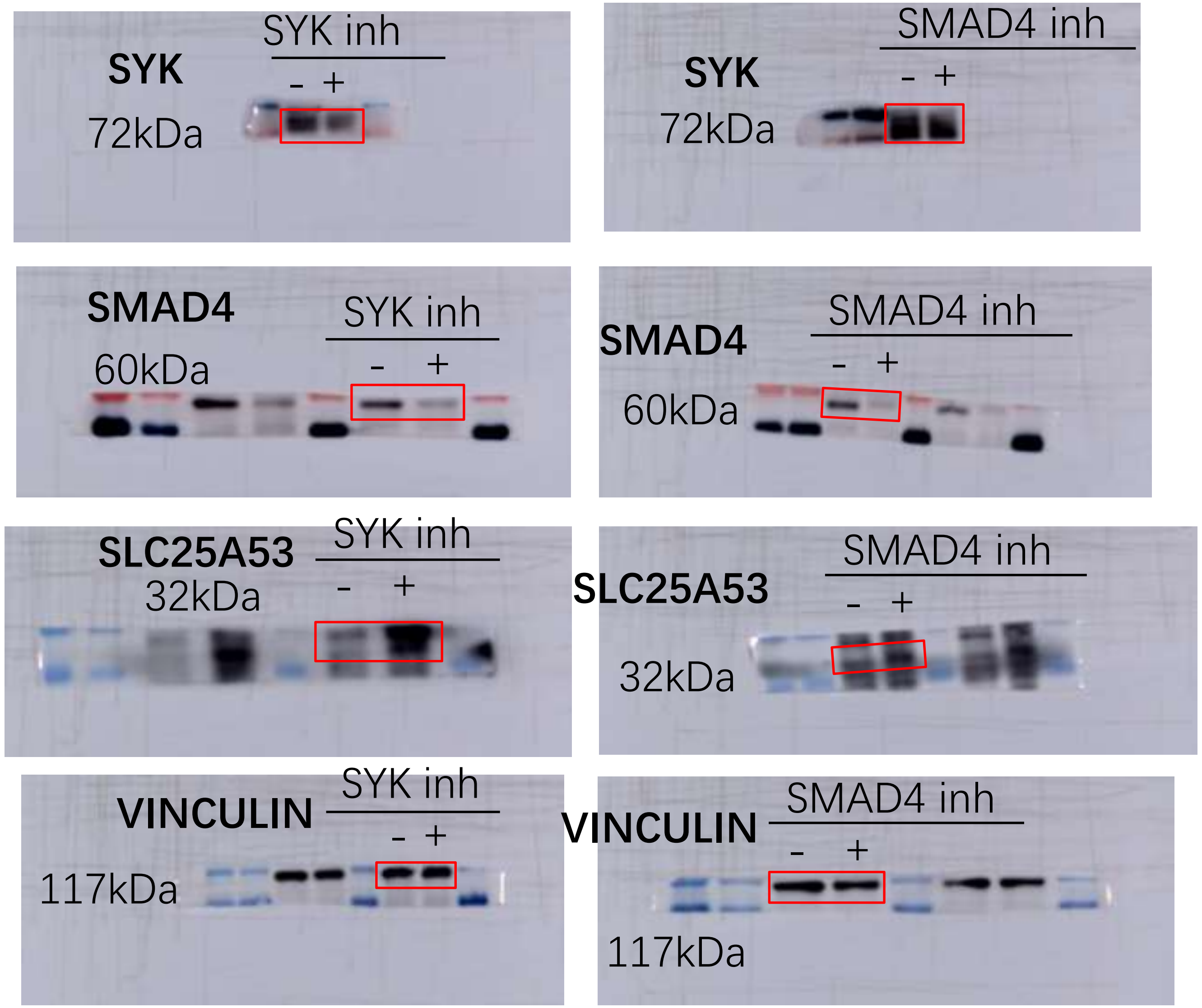
SYK
72kDa
SYK inh
- +
SYK
72kDa
SMAD4 inh
- +
SMAD4
60kDa
SYK inh
- +
SMAD4
60kDa
SMAD4 inh
- +
SLC25A53
32kDa
SYK inh
- +
SLC25A53
32kDa
SMAD4 inh
- +
VINCULIN
117kDa
SYK inh
- +
VINCULIN
117kDa
SMAD4 inh
- +

Figure4G

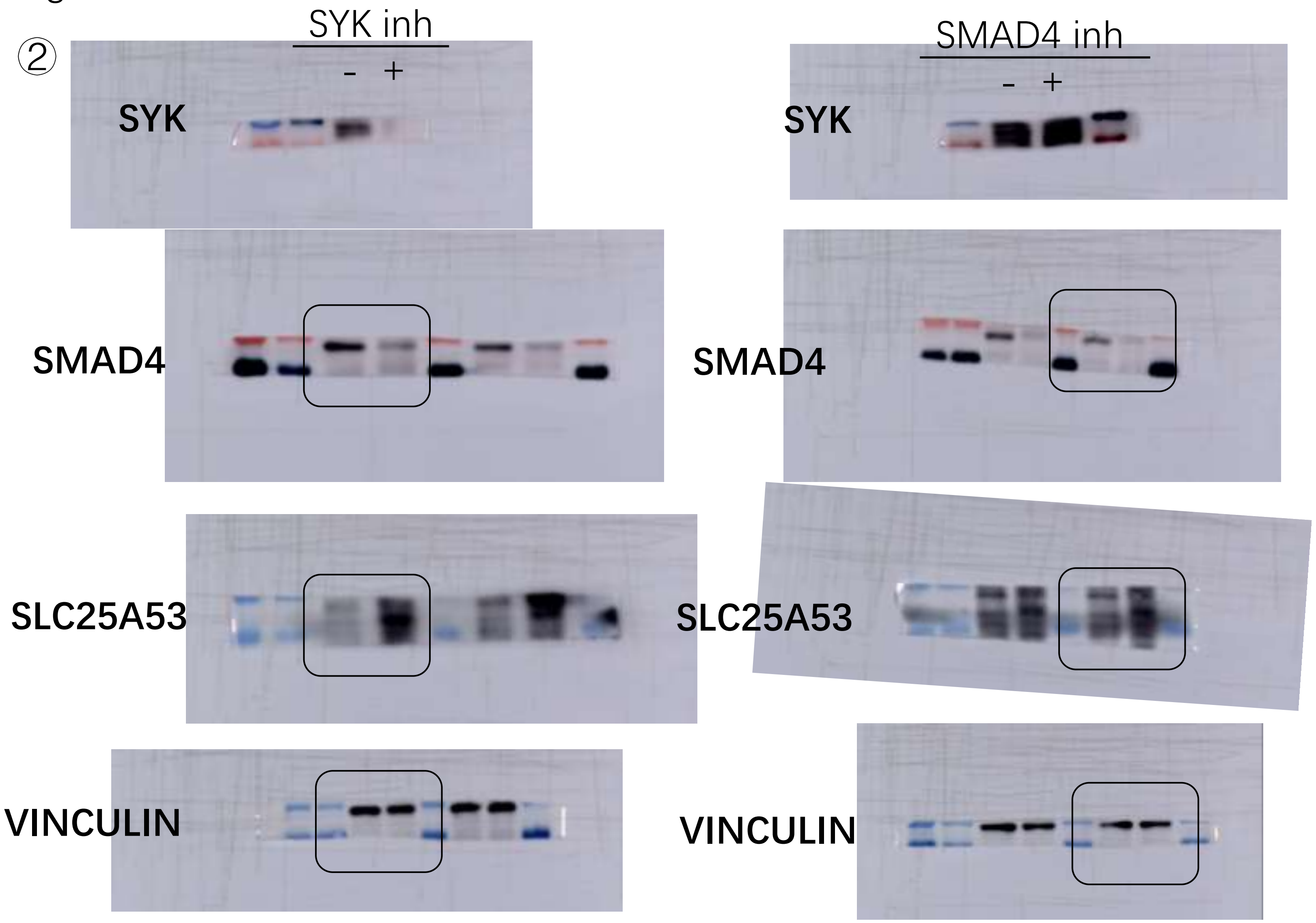
②
SYK inh
- +
SMAD4 inh
- +
SYK
SYK
SMAD4
SMAD4
SLC25A53
SLC25A53
VINCULIN
VINCULIN

Figure4G

③

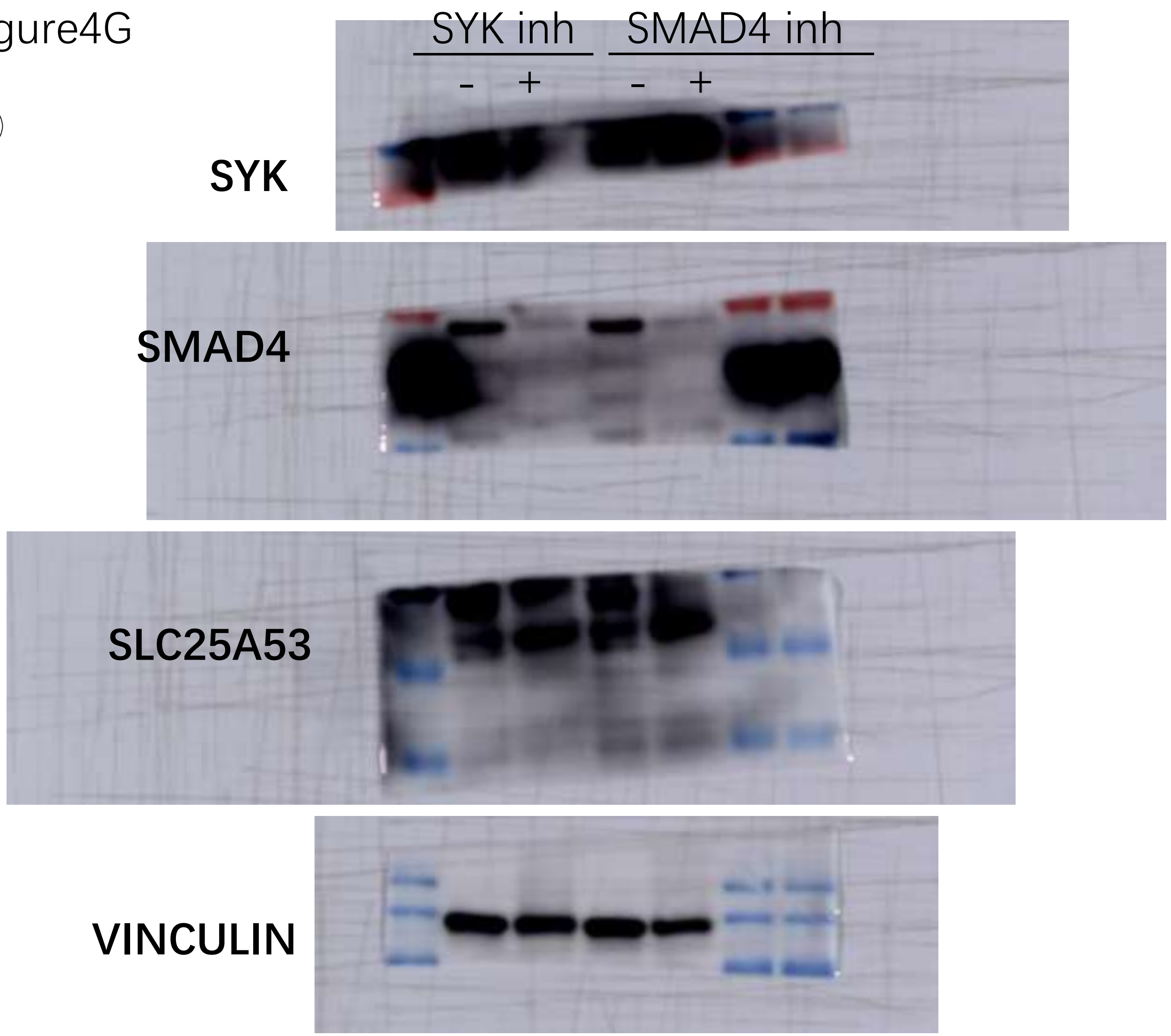

Figure6D ①-③

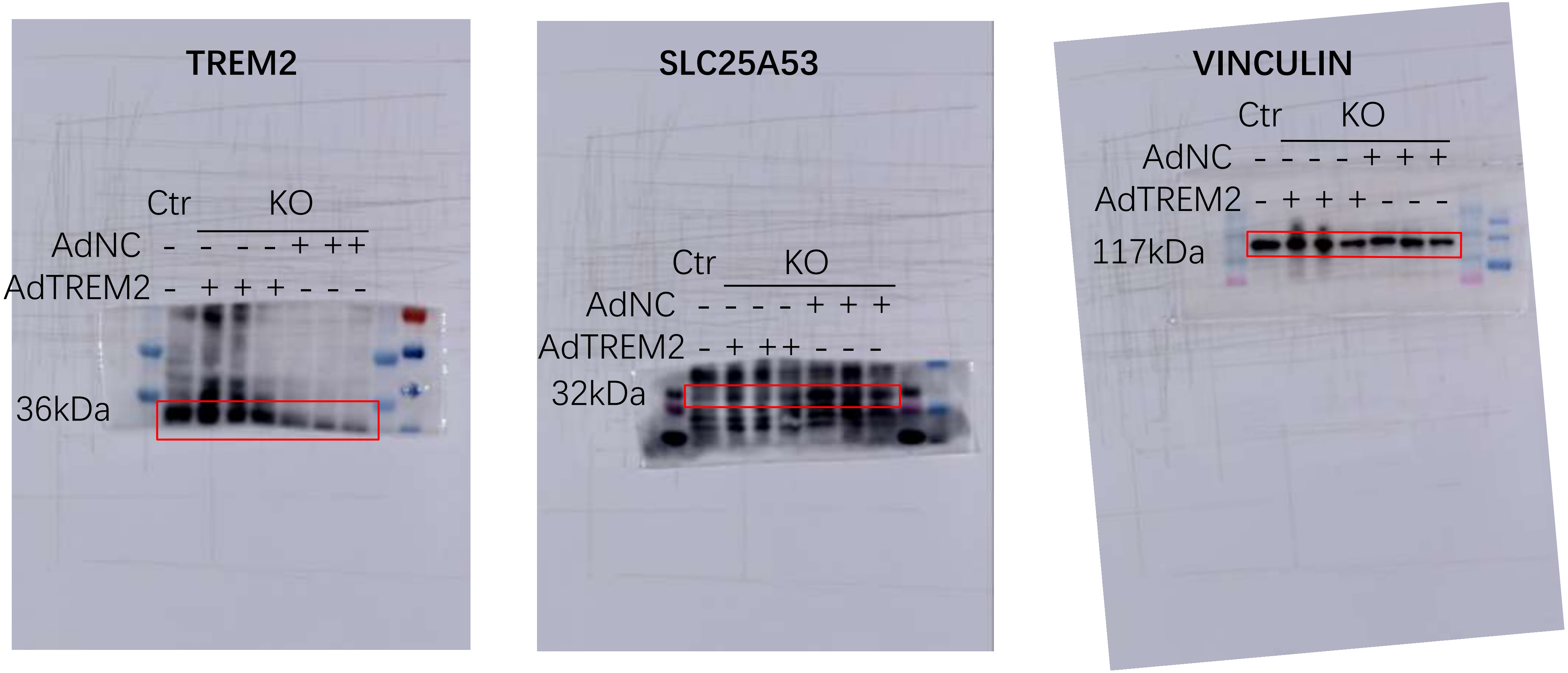
TREM2
Ctr KO
AdNC - - - - + + +
AdTREM2 - + + + - - -
36kDa
SLC25A53
Ctr KO
AdNC - - - - + + +
AdTREM2 - + + + - - -
32kDa
VINCULIN
Ctr KO
AdNC - - - - + + +
AdTREM2 - + + + - - -
117kDa

Supplementary Figure 1A ①-⑤

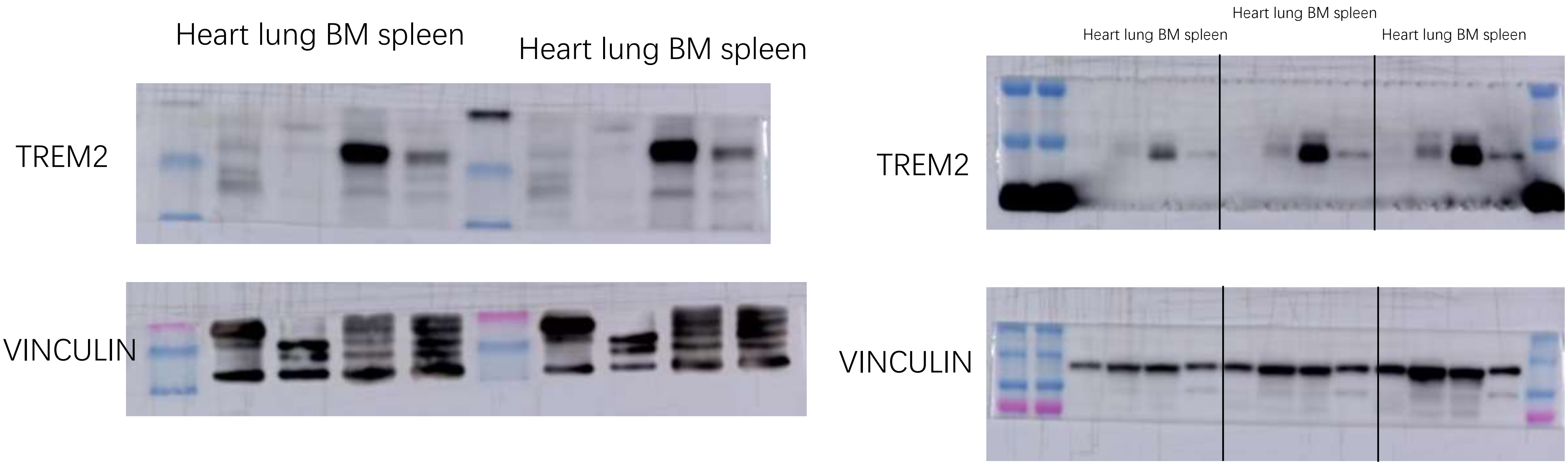

Supplementary Figure 1B ①-⑤

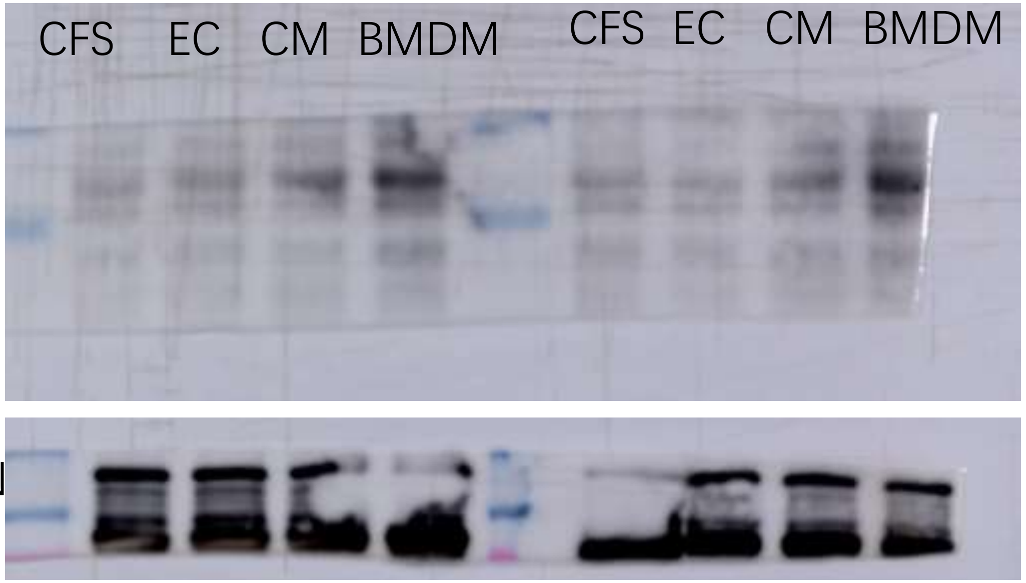

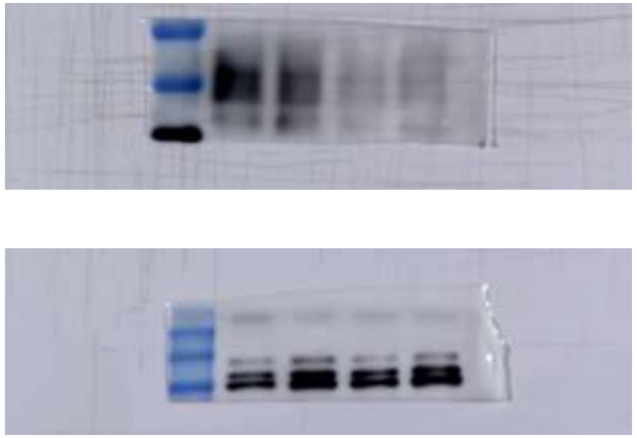

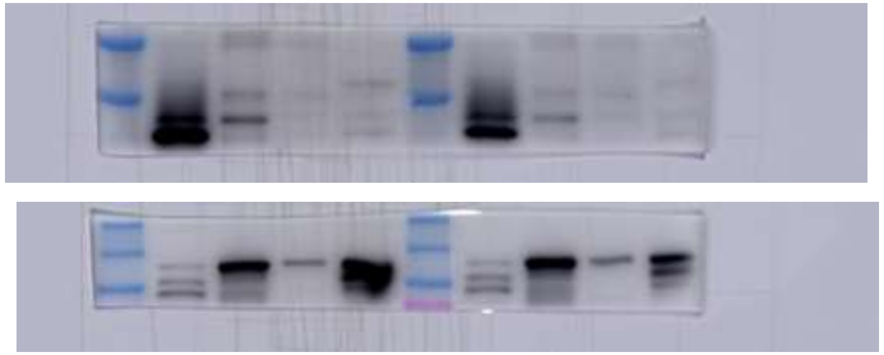

Supplementary Figure 3D ①-③

TREM2

VINCULIN

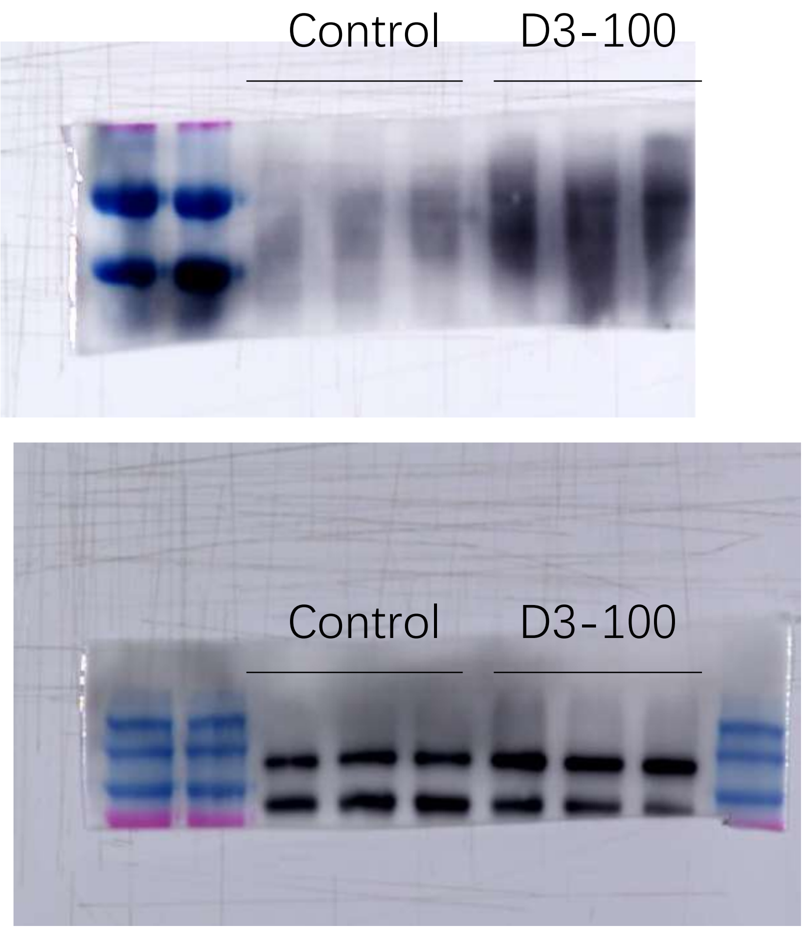

Supplementary Figure 6D ①-⑤

RAW

TREM2

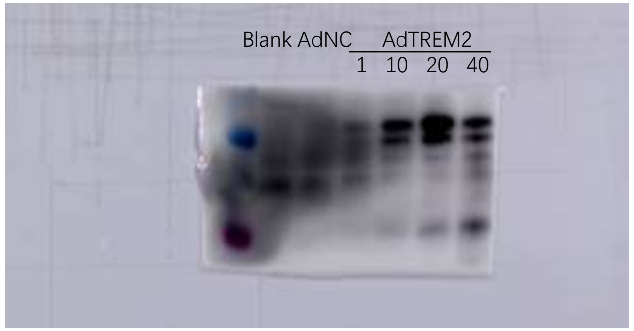

VINCULIN

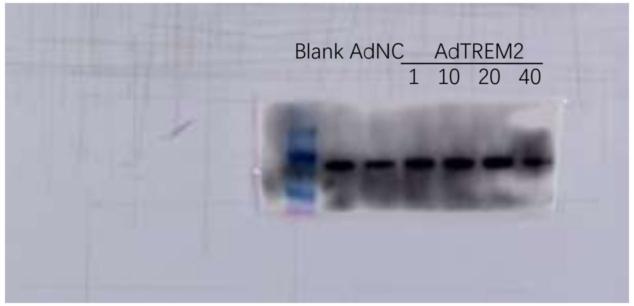

HEK293

TREM2

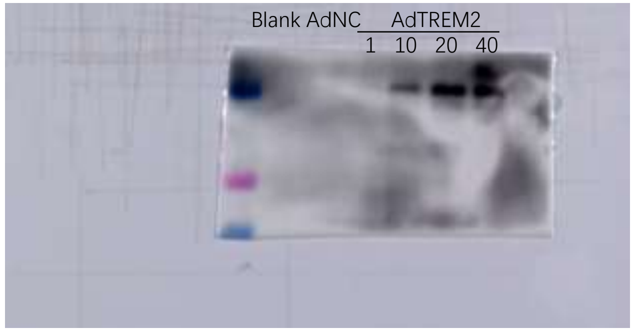

VINCULIN

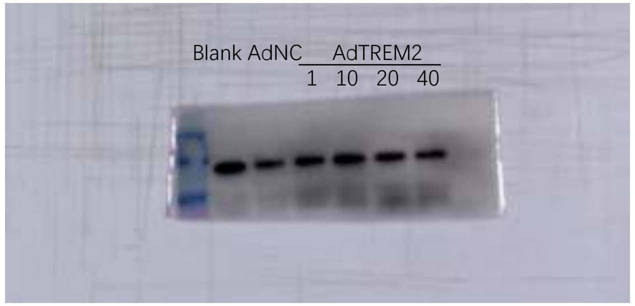

Supplementary Figure 8A ①-③

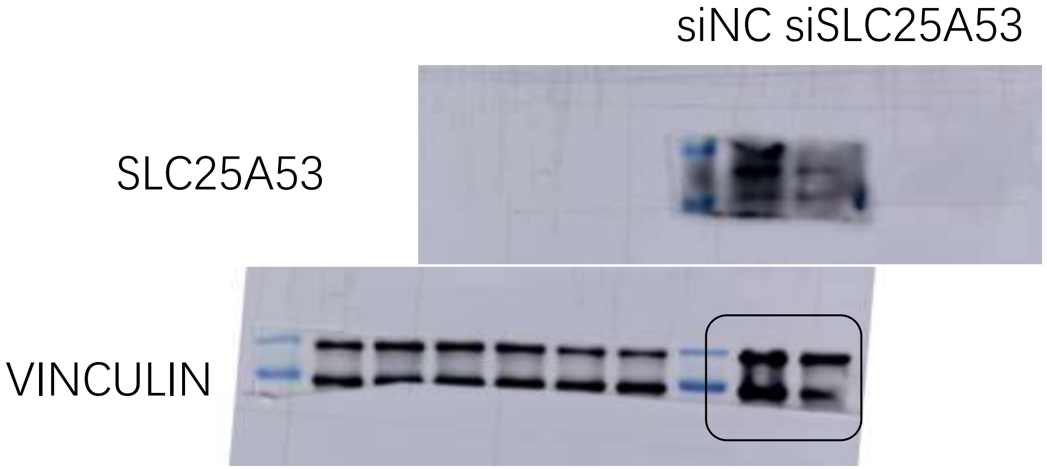

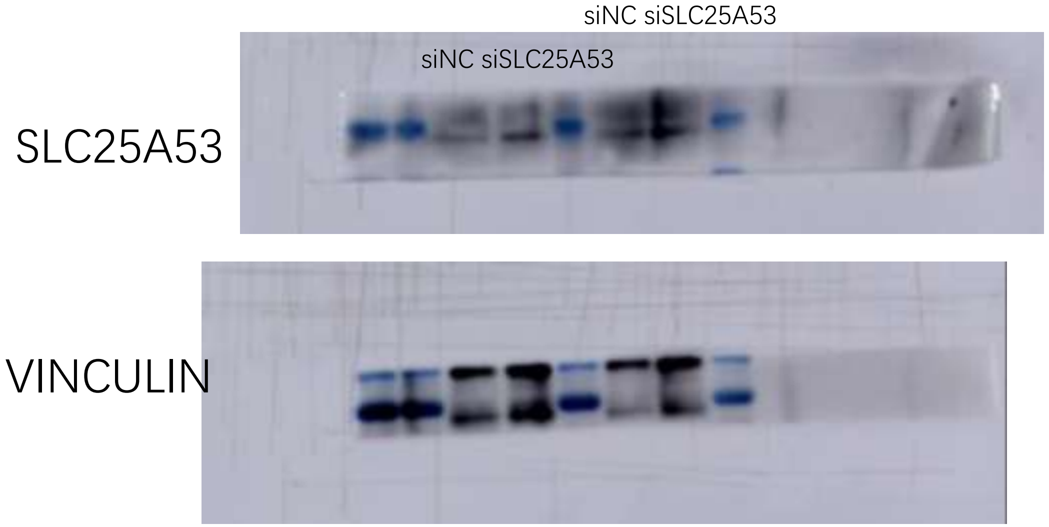

Supplementary Figure 9E ①-③

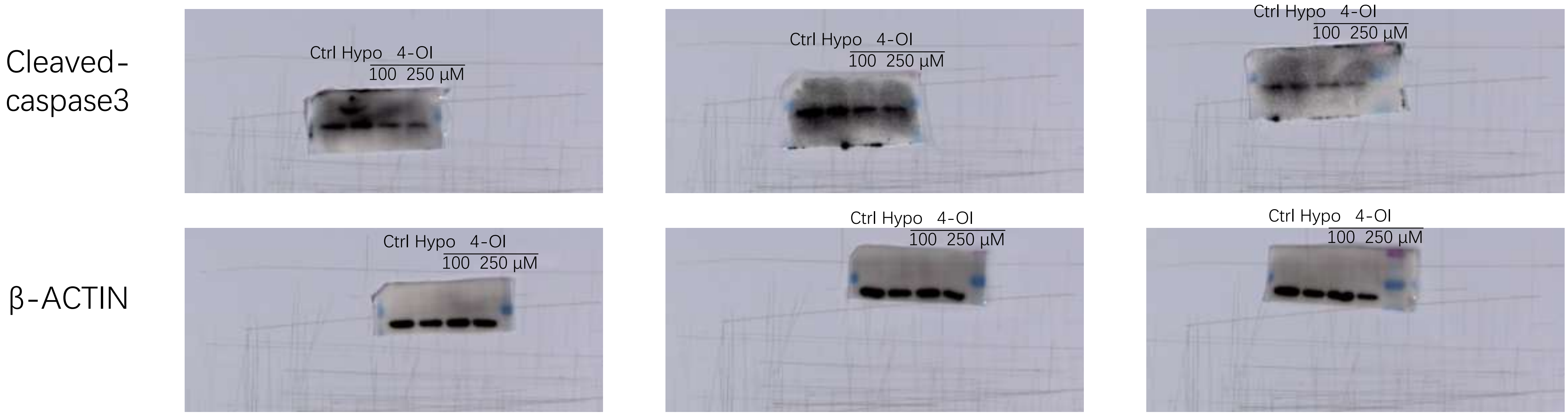

Supplementary Figure 11B ①-③

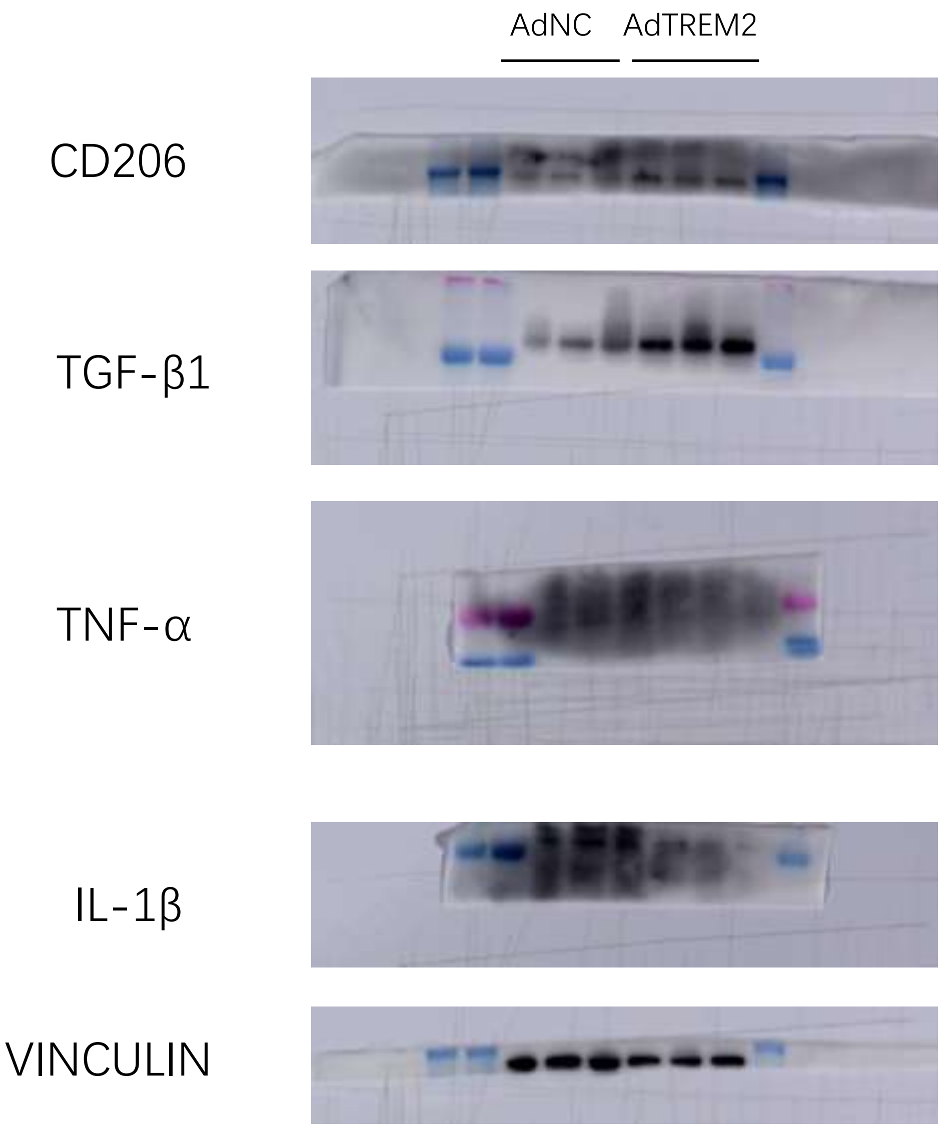

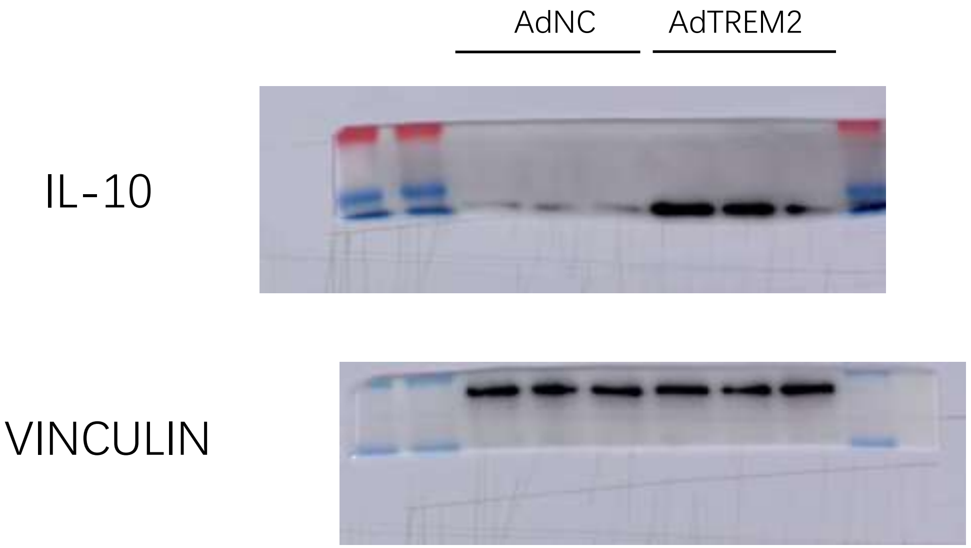

Supplement: Supplementary file 2 — Supplemental Material - Original Blots [file 41418_2023_1252_MOESM2_ESM.pdf]
